# Giant topological longitudinal circular photo-galvanic effect in the chiral multifold semimetal CoSi

**DOI:** 10.1038/s41467-020-20408-5

**Published:** 2021-01-08

**Authors:** Zhuoliang Ni, K. Wang, Y. Zhang, O. Pozo, B. Xu, X. Han, K. Manna, J. Paglione, C. Felser, A. G. Grushin, F. de Juan, E. J. Mele, Liang Wu

**Affiliations:** 1 Department of Physics and Astronomy, University of Pennsylvania, Philadelphia, PA 19104 USA; 2 Maryland Quantum Materials Center, Department of Physics, University of Maryland, College Park, MD 20742 USA; 3 Department of Physics, Massachusetts Institute of Technology, Cambridge, MA 02139 USA; 4 Max-Planck-Institut fur Chemische Physik fester Stoffe, 01187 Dresden, Germany; 5 Instituto de Ciencia de Materiales de Madrid, CSIC, 28049 Cantoblanco, Madrid Spain; 6 Department of Physics and Fribourg Center for Nanomaterials, University of Fribourg, Chemin du Musée 3, CH-1700 Fribourg, Switzerland; 7 Department of Physics, Indian Institute of Technology, Hauz Khas, New Delhi 110016 India; 8 Canadian Institute for Advanced Research, Toronto, ON M5G 1Z8 Canada; 9 University of Grenoble Alpes, CNRS, Grenoble INP, Institut Néel, 38000 Grenoble, France; 10 Donostia International Physics Center, P. Manuel de Lardizabal 4, 20018 Donostia-San, Sebastian Spain; 11 IKERBASQUE, Basque Foundation for Science, Maria Diaz de Haro 3, 48013 Bilbao, Spain

**Keywords:** Topological insulators, Nonlinear optics, Mid-infrared photonics, Infrared spectroscopy

## Abstract

The absence of mirror symmetry, or chirality, is behind striking natural phenomena found in systems as diverse as DNA and crystalline solids. A remarkable example occurs when chiral semimetals with topologically protected band degeneracies are illuminated with circularly polarized light. Under the right conditions, the part of the generated photocurrent that switches sign upon reversal of the light’s polarization, known as the circular photo-galvanic effect, is predicted to depend only on fundamental constants. The conditions to observe quantization are non-universal, and depend on material parameters and the incident frequency. In this work, we perform terahertz emission spectroscopy with tunable photon energy from 0.2 –1.1 eV in the chiral topological semimetal CoSi. We identify a large longitudinal photocurrent peaked at 0.4 eV reaching  ~550 μ A/V^2^, which is much larger than the photocurrent in any chiral crystal reported in the literature. Using first-principles calculations we establish that the peak originates only from topological band crossings, reaching 3.3 ± 0.3 in units of the quantization constant. Our calculations indicate that the quantized circular photo-galvanic effect is within reach in CoSi upon doping and increase of the hot-carrier lifetime. The large photo-conductivity suggests that topological semimetals could potentially be used as novel mid-infrared detectors.

## Abstract (web summary)

Quantized circular photogalvanic effect (CPGE) is predicted in chiral topological semimetals, but the experimental observation remains challenging. Here, Ni et al. observe a large topological longitudinal photocurrent in CoSi, which is much larger than the photocurrent in any other chiral crystals, indicating quantized CPGE within reach upon doping and increase of the hot-carrier lifetime.

## Introduction

Circular photo-galvanic effect (CPGE) exists only in gyrotropic crystals^1,2^. Its transverse component, where the current flows perpendicular to light propagation direction, is by far the most commonly observed. It has been recently measured in transition metal dichalcogenides^3^, topological insulators^4–6^ and Weyl semimetals^7–9^. In contrast, the longitudinal CPGE, where current flows parallel to light propagation direction, remains more elusive since its discovery in tellurium in 1979^10^.

**Fig. 1:**
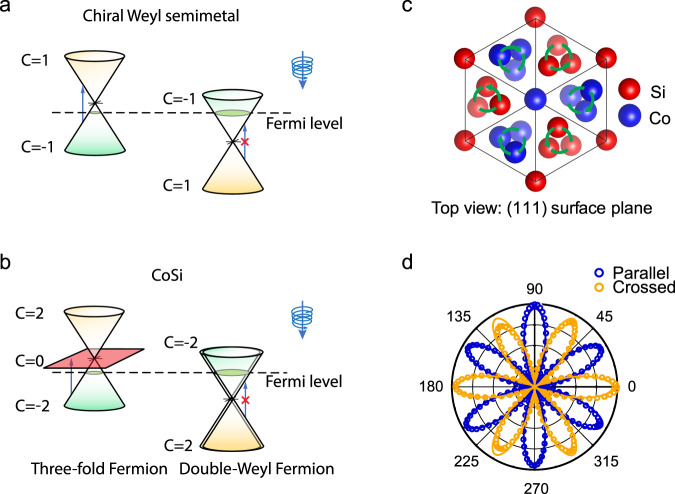
Schematic diagrams of the crystal and band structure of topological chiral semimetals, and the second harmonic generation on CoSi. **a** A chiral Weyl semimetal has two Weyl nodes with opposite monopole charges  ±1 at different energies. **b** Without spin-orbit coupling CoSi features a threefold node and a double Weyl node located at different energies and momenta. The three bands in the threefold fermions have Chern numbers +2, 0 and −2, respectively. When spin degeneracy is accounted for, the total charges at the two modes are  ±4. When circularly polarized light is incident on the sample, excitations around the right Weyl fermion (**a**) or the double Weyl fermion (**b**) are Pauli blocked, but excitations around the left Weyl fermion (**a**) or the threefold fermions (**b**) are allowed, generating a CPGE. **c** Schematic top view of CoSi (111) surface. The different transparency of Si and Co atoms indicates the different depth of each atom from the top plane. The green anticlockwise/clockwise circles indicate the chirality of the Co/Si atoms. **d** Second harmonic generation signal generated from CoSi (111) natural facet under normal incident laser pulses with a photon energy of 1.55 eV. Dots are experimental data from parallel and crossed configurations between incident and detecting linear polarization. Solid lines are the best fit.

In chiral topological semimetals, the longitudinal CPGE is particularly remarkable because it was recently predicted to be quantized^11–14^. These materials feature protected nodal crossings near the Fermi level, and because all mirror symmetries are broken, nodes with opposite chirality generically appear at different energies^15^ (see Fig. 1a, b), in contrast to mirror-symmetric Weyl metals, like TaAs with nodes at the same energy^16,17^. The existence of these nodes is protected by an integer topological charge *C*, which quantizes the longitudinal CPGE trace to *Cβ*_0_ where *β*_0_ = *πe*^3^/*h*^2^^11^. Chiral Weyl metals, where *C* = ±1 (Fig. 1a)^15^, are elusive. Nevertheless, separated topological nodes with degeneracies larger than two, known as multifold fermions, are demonstrated to exist in chiral crystals such as CoSi, RhSi and AlPt (with *C* = ±4)^12,18–23^ (Fig. 1b). Furthermore, the presence of cubic symmetry in these materials makes transverse CPGE vanishing and longitudinal CPGE isotropic with only one non-zero independent term, *β*_*xx*_, so that averaging over the three directions is not needed to measure the tensor trace *β* (*β* = 3*β*_*xx*_).

Several challenges remain to observe the quantized CPGE in chiral semimetals. In this family of materials, the presence of spin-orbit coupling leads to a splitting of the nodes, which can still display quantization but in a reduced frequency range determined by the strength of the spin-orbit coupling, for example as happens in RhSi^12–14,24,25^. Inter-band excitations between the spin-orbit split bands contribute to the non-quantized CPGE, a non-negligible effect in RhSi^14^. Therefore a small spin-orbit splitting is advantageous to observe the quantized CPGE in multifold materials^11,13^. In this work, we measure the CPGE in CoSi as its spin-orbit coupling is much smaller than in RhSi^12,19^.

We measure the CPGE by detecting radiated terahertz (THz) pulses emitted from the illuminated regions, a method with several advantages compared to DC current measurements ^8,9,24–27^. Firstly, detecting CPGE in a contact-less way avoids contact misalignment as we measure second harmonic generation to align the crystal axis. Secondly, the emitted THz pulse originates from a local illuminated region, and therefore thermal current and the non-local diffusion of photo-excited carriers to the contacts, typical of a DC current measurement, are not concerns^28^. Thirdly, in the process of THz emission, photo-carriers move at the band velocity and then recombine, which creates a time-dependent photo-current within the penetration depth. This time-dependent current radiates a THz pulse into free space, which in the far field is related to the first time-derivative of the surface current and is directly related with the quantized CPGE as the quantized quantity is the rate of the inject current, d*j*/d*t* = *iCβ*_0_, instead of the current *j* itself^29^. Note that optical rectification is generally at least two orders of magnitude smaller than the photocurrent in the resonant regime^27,30^. Therefore, we neglect the optical rectification effect in CoSi.

In this work, we have developed the capability to measure THz emission in the mid-infrared regime (0.20–0.48 eV) for the first time. We use it to measure CPGE in CoSi across a broad range of 0.2–1.1 eV. We identify a large longitudinal CPGE peaked at 0.4 eV reaching  ~ 550 μ A/V^2^. Comparing our measurements to first-principles calculations, we establish that the peak originates from topological band crossings, reaching 3.3 ± 0.3 in units of the quantization constant under the assumption of a constant hot-carrier lifetime. We develop a **k** ⋅ **p** model, including quadratic corrections to the dispersion of the nodal bands, and show that the location of the chemical potential can conspire to create a more complex frequency profile than it has been anticipated even in the spinless model. Our calculations also lay out the conditions to observe the quantized CPGE in CoSi in future experiments.

## Results

### Sample and second harmonic generation

The chiral crystal structure of CoSi seen from the (111) direction is depicted in Fig. 1c. As a first step, we pick up large homogeneous (111) natural facets by scanning second-harmonic generation (SHG) measurement^31^. To stimulate SHG, we focused light pulses centered at 800 nm under near-normal incidence to a 10-μm diameter spot on the sample and the second harmonic signal centered at 400 nm is measured. As shown in Fig. 1d, polar patterns of SHG are found as a function of the direction of the linear polarization of the incident light in the co-rotating parallel-polarizer (orange) and crossed-polarizer (blue) configurations. These patterns agree well with a fit with only one non-zero parameter based on the point group symmetry (see “Methods” section).

### Longitudinal CPGE in CoSi

**Fig. 2:**
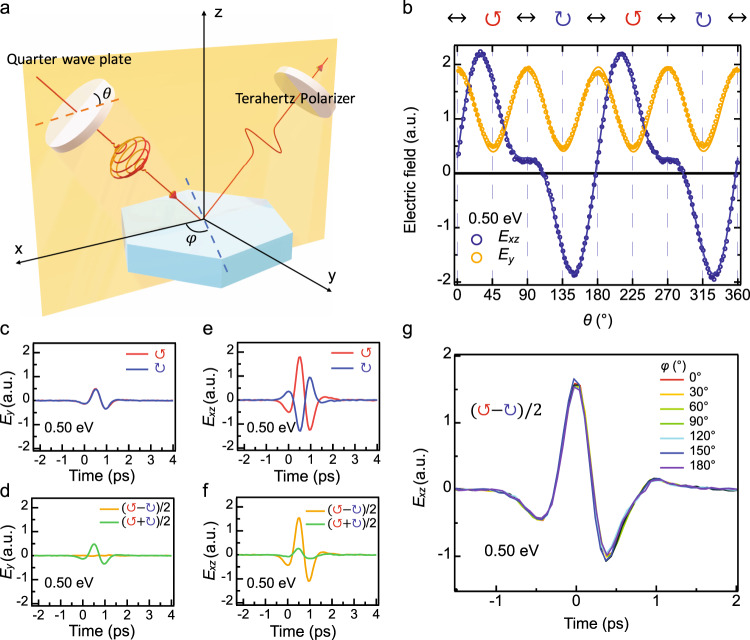
Schematic depiction of the CPGE experiment and experimental data on CoSi. **a** Schematic diagram of experimental setup. **b** A typical set of *xz* (in-plane) and *y* (out-of-plane) components of the peak of the emitted THz time trace as a function of the angle of the quarter-wave plate under light pulses centered at 0.50 eV. The open circles are experimental data and the lines are the best fit constrained by the crystal symmetry of CoSi. **c**–**f** A typical set of THz time traces of the out-of-plane (**c**) and in-plane (**e**) components under the left-handed and right-handed incident pulses at 0.50 eV. Curves in **d** and **f** describe the extracted contribution for CPGE ((*E*_↺_ − *E*_↻_)/2, orange) and LPGE ((*E*_↺_ − *E*_↻_)/2, green). **g** Nearly identical CPGE THz time traces at different sample azimuth angles *ϕ* at the incident photon energy of 0.50 eV.

Figure 2a shows schematically the measurement of the longitudinal CPGE. When circularly polarized light is incident on the sample, a current flows along the light propagation direction inside of the material. Under normal incidence, the current flows perpendicular to the surface, which prevents THz emission into free space from CPGE in the bulk^24,29^. THz emission does originate from the surface current density under oblique incidence^29^. See Supplementary Note 1 for more details. Therefore, in order to emit THz radiation into free space, we utilize off-normal incidence at 45 degrees as shown in Fig. 2a. An achromatic quarter-wave plate is used to control the polarization of the incident light. Terahertz wave components in *xz* and *y* direction are detected by using a THz polarizer before the ZnTe detector. Figure 2b, e shows the reversal of the polarity of the time trace of emitted THz electric field under left and right circularly-polarized light at the incident photon energy of 0.50 eV, which indicates the change of the direction of the photo-current under opposite helicity of circularly polarized incident light (i.e., the CPGE).

To confirm that the CPGE we observe is a longitudinal photocurrent, we studied the polarization dependence of the CPGE by rotating both the achromatic quarter-waveplate and the samples. The experimental geometry is shown in Fig. 2a, and we detect the emitted THz components in the incident plane, *E*_*xz*_(*t*), and perpendicular to the plane, *E*_*y*_(*t*). In Fig. 2b (orange) we show the peak value of the emitted THz field *E*_*y*_(*t*) at the incident photon energy of 0.50 eV as a function of the angle of the quarter-wave plate. The THz field under left and right circularly polarized light has the same direction and magnitude, which indicates no CPGE. The almost identical time traces of *E*_*y*_(*t*) with opposite circular polarizations are shown in Fig. 2c. The CPGE component (*E*_↺_(*t*) − *E*_↻_(*t*))/2 is zero within the detection sensitivity, as shown in Fig. 2d. (*E*_↺_(*t*) + *E*_↻_(*t*))/2 is the linear photo-galvanic effect (LPGE) component under circularly-polarized light (see Supplementary Note 1B for details).

In contrast, the in-plane THz field *E*_*xz*_(*t*) shows completely different polarization dependence as shown in Fig. 2b (blue). When the helicity of the circularly polarized light is reversed, the direction of the peak THz field in *E*_*xz*_(*t*) changes, and the waveform is shown in Fig. 2e. They are not simply the same curve with opposite signs because of a sizable LPGE contribution. Nevertheless, (*E*_↺_ − *E*_↻_)/2 is not zero in *E*_*xz*_(*t*) and relatively large compared to (*E*_↺_ + *E*_↻_)/2, as shown in Fig. 2f. The observation of a non-zero CPGE only in the incident *xz* plane is consistent with the longitudinal CPGE, where the current flows along the wave vector direction inside CoSi. This longitudinal CPGE is unchanged as we rotate the sample due to the cubic symmetry constraints, as shown for 0.50 eV incident photon energy in Fig. 2g. We also observed similar angle dependence at other photon energies. See Supplementary Note 2 for more details.

To quantify the longitudinal CPGE, we performed a symmetry analysis by fitting the angle dependence of the quarter-wave plate on *E*_*xz*_ and *E*_*y*_. The solid lines in Fig. 2b are the best fit to the functions determined by the crystal symmetry, ${E}_{y}(\theta )={B}_{1}\sin (4\theta )+{C}_{1}\cos (4\theta )+{D}_{1}$ and ${E}_{xz}(\theta )=A\sin (2\theta )+{B}_{2}\sin (4\theta )+{C}_{2}\cos (4\theta )+{D}_{2}$, where the coefficients *A*, *B*_1_, *B*_2_, *C*_1_, *C*_2_, *D*_1_, *D*_2_ are determined by the CPGE and LPGE conductivity (see Supplementary Note 1B for details). Both curves are fitted simultaneously with the same fit weights. The $\sin (2\theta )$ term describes the CPGE while $\sin (4\theta )$, $\cos (4\theta )$ and the constant terms describe the LPGE. The symmetry analysis shows that the out-of-plane component *E*_*y*_ does not contribute to the CPGE, while the in-plane component *E*_*xz*_ dominates the CPGE.

### CPGE spectrum in CoSi

After confirming the longitudinal direction of the CPGE, we now study the amplitude of the CPGE current inside the sample at different incident photon energies. We use circularly-polarized laser pulses with a duration 50–100 fs and a tunable incident photon energy from 0.2 eV to 1.1 eV to generate a CPGE inside of the sample. For the first time, we detected THz emission with incident photon energy below 0.5 eV, comparing with previous measurements^8,9,24,26,27^. In order to convert the detected THz electric field into the CPGE current inside the sample, we measured a benchmarking ZnTe sample at the same position at each wavelength immediately after measuring CoSi. ZnTe is useful as a benchmark due to its relatively flat frequency dependence on the electric-optical sampling coefficient for photon energy below the gap^32^. See Supplementary Note 1F and Supplementary Fig. 3 for the raw data. We first convert the collected THz electric fields on CoSi and ZnTe from the time domain to the frequency domain by a Fourier transformation. By taking the ratio of the two Fourier transforms of the electric fields and considering the Fresnel coefficient, refractive indices and penetration depth, we obtain the ratio between the CPGE response of CoSi and the optical rectification of ZnTe. The use of ZnTe circumvents assumptions regarding the incident pulse length, the wavelength dependent focus spot size on the sample, and the calculation of collection efficiency of the off-axis parabolic mirrors (see Supplementary Note 1C for details).

**Fig. 3:**
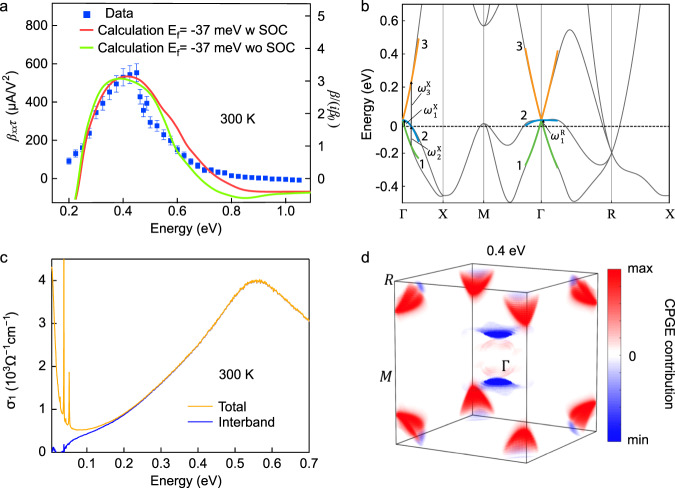
Room tempreture CPGE spectrum, optical conductivity and band structure for CoSi. **a** Measured second-order CPGE photo-conductivity (*β*_*xx*_*τ*) as a function of incident photon energy, and ab-intio calculations of the CPGE current with and without spin-orbit coupling at room temperature. **b** The band structure of CoSi without spin-orbit coupling. We define zero energy at the threefold node at the *Γ* point. The double Weyl node at the *R* point is at −185 meV. The dashed horizontal line indicates the chemical potential *E*_*f*_ = −37 meV in our sample, moderately lower from that obtained by DFT (*E*_*f*_ = −20 meV). The band structure of the **k** ⋅ **p** model is shown in green (band 1), blue (band 2), and orange (band 3) obtained by fitting the ab-intio band structure (black) up to quadratic corrections. For the *Γ*−*X* direction we define ${\omega }_{1}^{X}$, ${\omega }_{2}^{X}$, and ${\omega }_{3}^{X}$ as the minimum energy that allows transitions from band 1 to band 2, the maximum energy that allows transition from band 1 to band 2 and the minimum energy that allows transitions from band 2 to band 3, respectively. We define in the same way ${\omega }_{1}^{R}$ in the *R* direction (${\omega }_{2,3}^{R}$ fall outside the applicability of the quadratic model). **c** Total (gold) and interband (blue) optical conductivity of CoSi at 300 K. **d** Momentum resolved contributions to the CPGE peak at 0.4 eV in the red curve in **a**.

The CPGE follows $j(\Omega )=\frac{{\beta }_{xx}}{i\Omega +1/\tau }{E}_{0}^{2}(\Omega )$, where Ω is the THz frequency and *τ* is the hot-carrier lifetime. When the hot-carrier lifetime satisfies *τ* ≪ 1/Ω, $j\approx {\beta }_{xx}\tau {E}_{0}^{2}$. This is the case for the current experiment as *j*(Ω) depends weakly on Ω. (See Supplementary Fig. 6.) The second-order photo-conductivity plotted in Fig. 3a was an average value of the CPGE over the frequency range of 0.5–2.0 THz in Supplementary Fig. 6, which should also be the DC limit. When *τ* is much longer than the pulse width, which is in the quantization regime, ${\rm{d}}j/{\rm{d}}t={\beta }_{xx}{E}_{0}^{2}$. When 1/*τ* is comparable to Ω, the CPGE conductivity, $\frac{{\beta }_{xx}}{i\Omega +1/\tau }$, will have strong frequency dependence in the THz regime, which will enable the extraction of *β*_*xx*_ and *τ* separately. Anticipating our theory analysis, we note that the CPGE spectrum is determined by the only symmetry-independent non-zero CPGE response tensor *β*_*xx*_, which is a photocurrent rate multiplied by the hot-carrier lifetime *τ*. The measured CPGE photocurrent per incident field squared as a function of frequency, which we will denote as the CPGE spectrum, is shown in Fig. 3a for room temperature, showing a peak value of  ~550 μ A/V^2^ at 0.4 eV. The CPGE spectrum peak value is much larger than the photo-galvanic effect in any chiral crystals reported in the literature^33^, BaTiO_3_^34^, single-layer monochalcogenides^34,35^, the colossal bulk-photovoltaic response in TaAs^36^ and RhSi in the same space group^24,25^.

### First-principle calculation

Next, we address the relationship between the large photo-conductivity peak and the multifold fermions near the Fermi level shown in Fig. 3b. In Fig. 3a, we show our ab-initio calculations of the CPGE for CoSi with and without spin-orbit coupling (SOC) at room temperature and at a chemical potential *E*_*f*_ crossing the flat hole band, as indicated by the dashed line in Fig. 3b (see “Methods” section and Supplementary Note 4). They quantitatively reproduce the experimental data across a wide frequency range. The SOC splitting,  ≈20 meV at the *Γ* point node, determines the finer structure in the optical response^37^.

To match the first-principles calculations with the CPGE spectrum, we considered *β*_*xx*_, which is related to the CPGE trace by *β* = 3*β*_*xx*_ due to cubic symmetry^11,12^. Note that *β*_*xx*_ is directly calculated from the band structure at certain chemical potential. It is plotted in Fig. 3a times *τ*, the only free parameter, which was determined by matching the calculated peak width and magnitude to the CPGE data. This self-consistent constraint is satisfied with a broadening *ℏ*/*τ* ≈ 38 meV at 300 K. As shown from the right *y* axis of Fig. 3a, the CPGE trace *β* reaches 3.3 (±0.3) in units of the quantization constant *β*_0_.

For frequencies below 0.6 eV, all the interband excitations on CoSi occur in the vicinity of its multifold bands at the nodes *Γ* and *R*^37^. We conclude this from the optical conductivity on CoSi at 300 K that is shown in Fig. 3c. With the subtraction of the Drude response and the four phonon peaks, the interband contribution has a kink at  ~0.2 eV that separates two quasi-linear regimes. The details of the theoretical and experimental studies could be found in ref. ^37^. Below 0.2 eV, the interband excitations involve the threefold fermion at the *Γ* point, while the excitations near the double Weyl fermion at *R* become active only above 0.2 eV^37^. The main contribution to the peak around 0.6 eV comes from the saddle point *M*^37^. In addition to the optical conductivity, our momentum resolved calculation (Fig. 3d) for the CPGE peak at 0.4 eV reveals that it originates from these multifold fermions only, which contribute with opposite signs to the CPGE current. Therefore, this giant CPGE peak has a purely topological origin, although it is not quantized due to the sum of contributions of two kinds of multifold fermions and the quadratic contributions to the energy bands.

**Fig. 4:**
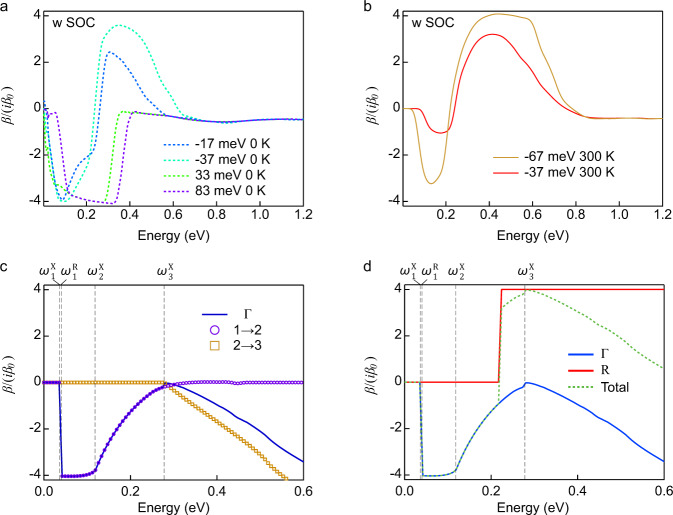
CPGE calculations for CoSi. **a**, **b** CPGE current obtained by ab-intio calculations corresponding to the CoSi band structure with spin-orbit coupling at different chemical potentials at **a**, 0 K with 5 meV broadening and **b** 300 K with 38 meV broadening. **c**, **d** CPGE current calculation from the **k** ⋅ **p** model, with parameters (*v*, *a*, *b*, *c*) = (1.79, 1.07, −1.72, 3.26). **c** The contributions to the CPGE current from transitions near the *Γ* point are shown by open purple circles for transitions from band 1 to 2 and as open gold squares for transitions form band 2 to 3. A solid blue line shows the sum of the contributions form transitions from 1 to 2 and from 2 to 3, which is the total CPGE contribution from the *Γ* point. **d** The contribution from the *R* point to the CPGE is shown by a step of 4 in red. The total contribution to the CPGE from the *Γ* and the *R* point is shown in green. A quantized dip/plateau is observed when the frequency is between ${\omega }_{1}^{R}$ and ${\omega }_{2}^{X}$, which allow transitions from band 1 to band 2 only. The dip is determined by transitions around *Γ* only, while the peak has contributions from excitations near the *Γ* and *R* points.

The position of the chemical potential is crucial to relate the CPGE to quantization. Our ab-initio calculations, supported by our low-energy analysis below, reveal that the CPGE shows a dip-peak structure as the one of Fig. 4a, b) when *E*_*f*_ is below the *Γ* node (*E*_*f*_ < 0) in our sample. Such Fermi energy is consistent with recent quasi-particle interference^38^ and linear optical conductivity experiments^37^. Note that this dip-peak structure was clearly observed recently in RhSi as the energy splitting between the nodes at the *Γ* and *R* in RhSi is around twice larger than CoSi so that the sign change in CPGE is pushed to around 0.4 eV in RhSi^25^. The dip-peak structure for *E*_*f*_ < 0 is also produced by using a four-band tight binding model for CoSi^13^ (see Supplementary Note 5). As shown in Fig. 4a, the dip reaches the quantized value of 4*β*_0_ at low temperatures in the clean limit, and remains quantized for hot carrier lifetime broadening up to 5 meV at 100 meV photon energy (see Supplementary Note 4 and Supplementary Fig. 7 for details.). The quantization of the dip is determined by the threefold fermion at the *Γ* point as the vertical excitations at the *R* point are Pauli blocked below 0.2 eV^37^. Therefore, the CPGE will not be quantized in the current sample at low temperature in the photon energy range of 0.2–1.1 eV as the peak around 0.4 eV after the dip appears non-universal in general due to contributions from both nodes at *Γ* and *R* (see see *E*_*f*_ = −37 meV curve in Fig. 4a). However, if *E*_*f*_ is decreased further to lie close to the *R* point, this peak can reach 4*β*_0_ at room temperature even with a broadening of 38 meV (see *E*_*f* _= −67 meV curve in Fig. 4b). As discussed below, this peak originates from the double Weyl fermion at the *R* point, and it is enabled by an accidental window of vanishing CPGE contribution from the *Γ* point. Finally, we note that electron-electron interactions can also correct the quantized value, as occurs for chiral Weyl semimetals^39^. While it is currently unknown how relevant these corrections are for multifold fermions, the large hole and electron pockets at *Γ* and *R* in CoSi suggest that screening should be strong and therefore interactions should have a small effect. The good agreement of our model calculations with the data, shown in Fig. 3a, is also consistent with this point of view. Experimentally, from the optical conductivity measurements we estimate a relative dielectric constant *ϵ*_1_ of the order of −2500 at 300 K and −10,000 at 10 K, further supporting a normal metallic behavior with very large screening of interactions. Also, specific heat measurements on CoSi also showed that it is a weakly correlated semimetal, as evidenced by a normal metallic Sommerfeld constant^40^. Because of these reasons interactions are neglected in this work.

### **k** ⋅ **p** model

To understand the origin of the dip-peak structure, it is necessary to describe the curvature of the middle band. To this end we derived a low-energy **k** ⋅ **p** type model keeping symmetry-allowed terms up to quadratic order in momentum **k**. The resulting Hamiltonian reads

1 $$H= v{\bf{k}}\cdot {\bf{S}}+\left(\begin{array}{ccc}{c}_{1}{k}^{2}-2c{k}_{z}^{2}&b{k}_{y}{k}_{z}&b{k}_{z}{k}_{x}\\ b{k}_{y}{k}_{z}&{c}_{1}{k}^{2}-2c{k}_{x}^{2}&b{k}_{x}{k}_{y}\\ b{k}_{z}{k}_{x}&b{k}_{x}{k}_{y}&{c}_{1}{k}^{2}-2c{k}_{y}^{2}\end{array}\right),$$

where **S** is the vector of spin-1 matrices, and *k* = ∣**k**∣. We fixed its coefficients *v*, *b*, *c*, and ${c}_{1}=\frac{1}{3}(3a+2c)$ with a fit to the band structure shown in Fig. 3b around the *Γ* point. The second term includes three out of the four symmetry-allowed quadratic terms because the fourth has a negligible effect on the CPGE (see Supplementary Note 6 for details). The energies expanded to second order in momentum for the three bands are plotted as colored lines in Fig. 3b. The coefficients *b* and *c* determine the curvature in the *Γ*−*X* and *Γ*−*R* directions, respectively. For the *R* point bands, we use a spin-degenerate double Weyl model that has a step increase in the CPGE current by 4*β*_0_ when excitations at *R* are allowed in Fig. 4d^12^.

The possible optical transitions in the band structure near the *Γ* point are illustrated in Fig. 3b. We label the bands with increasing energies as 1, 2, 3. For *E*_*f*_ above the threefold node, the only possible transition is from bands 2 to 3. As the frequency increases, this transition becomes active and yields a monotonically increasing joint density of states (JDOS)^13^. As shown in Fig. 4c, for *E*_*f*_ below the node, however, two types of transitions contribute: 1 to 2 and 2 to 3. The first transition from band 1 to band 2 (open purple circles) is active for a small range of energies, and then decays to zero. The second transition from band 2 to band 3 (open gold squares) only starts picking up at larger frequencies, leaving a dip in the JDOS and, therefore, a dip in the CPGE (solid blue line). The different frequencies where the transitions become active or inactive are labeled in Figs. 3b, 4c, d. Figure. 4d show that when we add the contributions from the threefold fermions at *Γ* and double Weyl fermion at *R*, the existence of the dip from the threefold fermions leads to the dip-peak structure observed in the ab-initio calculations, only when *E*_*f*_ is below the threefold node. In the **k** ⋅ **p** model, we also show that while the dip is universally quantized, the peak is not because of the incomplete transitions from *Γ*. Note that the quantization of the peak not only depends on the *Γ* contribution but it also may be altered by the quadratic dispersion of the double Weyl fermion when it fully contributes to CPGE. However, as shown in Fig. 4b, decreasing *E*_*f*_ further could be used to diminish the contribution from the threefold fermions at around 0.4 eV and reveal the quantization due to the *R* point (see Supplementary Note 4 for details).

## Discussion

By studying the CPGE in the chiral topological semimetal CoSi we found a large longitudinal photo-conductivity in the mid-infrared regime, which has a purely topological origin linked to the existence of multifold fermions in this material. CoSi could potentially be used as a new mid-infrared detector based on a topological mechanism if the hot-carrier lifetime could be increased to around 1 ps, as observed in other semimetals^41^. Moreover, our theory suggests that a quantized CPGE is within reach in CoSi by several means. With the chemical potential below the threefold node, the very narrow quantized plateau around 100 meV corresponding to the *Γ* node could be accessible at low temperatures if the hot-carrier lifetime increases by one order of magnitude. Also, electron doping the *E*_*f*_ above the threefold node will result in a wider quantized plateau over 100–350 meV from the *Γ* node corresponding to the dip we calculate at low temperatures also if the hot-carrier lifetime increases by one order of magnitude. A quantized plateau around 0.4 eV corresponding to the *R* node can be revealed at *T* = 300 K by hole-doping, even with a similar short hot carrier lifetime as that of our current sample. We expect that these possibilities, opened by our work, motivate further effort on crystal growth with longer hot carrier lifetime and different doping, as well as time-resolved measurements in the mid-infrared regime to probe the hot carrier dynamics. The methods developed in our work could also be applied to other chiral topological semimetals^18,42^ to realize the quantized CPGE.

## Methods

### Crystal growth

High quality CoSi single crystals were prepared by a high temperature flux method with tellurium as flux. Cobalt pieces (Alfa Aesar 99.98%), silicon pieces (Alfa Aesar 99.999%), and tellurium lumps (Alfa Aesar 99.99%) with the molar ratio of 1:1:15–20 were set in an alumina crucible and then sealed in a fused silica ampule in around 0.8 atm argon environment. The ampule was heated to 1100 ^∘^C with a speed of about 150 ^∘^C/h. After soaking at 1100 ^∘^C for 10 h, the ampule was cooled down to 700 ^∘^C at the rate of 2 ^∘^C/h, and the excess flux was centrifuged out at that temperature to get several single crystals with large (111) facet^43^. Crystals larger than 2 ×  2 mm were picked for THz emission experiments. We also performed a spatial SHG scanning of the sample and found an homogeneous signal.

### Second harmonic generation fit

For CoSi (111), the fits are:

2 $${I}_{{\mathrm{parallel}}}(\theta )=\frac{1}{6}| {\chi }_{xyz}^{(2)}{|}^{2}{\left({\cos}^{3}\theta -3\,{\cos} \,\theta \sin ^{2}\theta \right)}^{2}.$$

3 $${I}_{{\rm{crossed}}}(\theta )=\frac{1}{6}| {\chi }_{xyz}^{(2)}{| }^{2}{\left({\sin }^{3}\theta -3\,{\cos }^{2}\theta \sin \theta \right)}^{2}.$$

${\chi }_{xyz}^{(2)}$ is the only non-zero SHG tensor element in CoSi. *θ* is the angle between the incident polarization and the [1, 1, −2] axis.

### Terehertz emission spectroscopy

A laser beam from a Ti:sapphire amplifier (center photon energy 1.55 eV, repetition rate 1 kHz, duration  ~35 fs) was split by a beam splitter into pump and probe beams. On the pump side, an optical parametric amplifier is used to convert the photon energy to 0.47–1.1 eV (pulse duration 40–70 fs), and a different frequency generation is used to further convert photon energy to 0.20–0.48 eV (pulse duration 70–110 fs). The laser beams were then focused by a 40-cm BaF_2_ lens or a 40-cm germanium lens onto the sample with a diameter of 1 mm under 45 degree angle of incidence. A typical pump power of 15 μJ per pulse was used, which falls into the linear response range. The emitted THz wave was collected by an off-axis parabolic mirror (OAP) and focused by another OAP onto an electro-optic (EO) crystal, ZnTe (110). The probe beam was co-propagating with the THz wave into the EO crystal to detect the THz electric field using EO-sampling method^29^. All of the measurement were performed in a dry-air environment with relative humidity less than 3% to avoid water absorption. To control the polarization of pump pulses, a quartz-MgF_2_ achromatic quarter-wave plate (600–2700 nm, retardance error ≤ *λ*/500) and a MgF_2_ achromatic quarter-wave plate (2500–7000 nm, retardance error ≤ *λ*/100) were used. A THz wire-grid polarizer was used to extract out-of-plane (*E*_*y*_) and in-plane (*E*_*xz*_) components of THz electric field. A benchmarking crystal ZnTe (110) was used as a standard candle to extract the photo-galvanic response from CoSi. Both crystals were mounted on a computer-controlled motor to reliably change the position. For each incident photon energy, measurement of CoSi was immediately followed by ZnTe to avoid long-term fluctuation of laser power. By comparing the THz electric field of CoSi and ZnTe in frequency domain, the photo-galvanic response of the CoSi crystal could be obtained (see Supplementary Note 1 for details).

### First principle calculation

To calculate the CPGE current, we obtain the density-functional theory (DFT) Bloch wave functions from the Full-Potential Local-Orbital program (FPLO)^44^ within the generalized gradient approximation (GGA)^45^. By projecting the Bloch wave functions onto Wannier functions, we obtain a tight-binding Hamiltonian with 104 bands from 3*d*, 4*s*, 4*p* orbitals of Co and 3*s*, 3*p*, orbitals of Si, which we use for efficient evaluation of the CPGE photocurrent.

To implement the CPGE integrals in Eq. (4), the Brillouin zone was sampled by *k*-grids from 200 × 200 × 200 to 960 × 960 × 960^46^. Satisfactory convergence (less than 2% change) was achieved for a k-grid of size 400 × 400 × 400. The temperature dependence is implemented by the Fermi-Dirac distribution function and we also include a hot-carrier lifetime broadening factor (see Supplementary Note 4 for details). CoSi is in space group *P*2_1_3 (*#*198), with point group 23 (*T*). Owing to the two-fold glide rotation symmetry *s*_2*x*_, *s*_2*y*_, *s*_2*z*_, only diagonal tensor elements are nonzero, and the *C*_3_ rotation symmetry further leads to a single independent component *β*_*xx*_ = *β*_*yy*_ = *β*_*zz*_. We carefully checked the symmetry of numerically calculated tensor elements with the tensor shape given by lattice symmetry and found the errors to be within 10^−6^. The full circular photo-galvanic effect tensor is given by

4 $${\beta }_{ab}(\omega )=	\frac{i\pi {e}^{3}}{4\hslash }{\int}_{{\rm{BZ}}}\frac{{\rm{d}}{\bf{k}}}{{(2\pi )}^{2}}\mathop{\sum }\limits_{n \,{> }\,m}{\epsilon }^{bcd}{f}_{nm}\\ 	 \times {\Delta }_{mn}^{a}{\rm{Im}}[{r}_{nm}^{d}{r}_{mn}^{c}]{{\mathcal{L}}}_{\tau }({E}_{nm}-\hslash \omega ),$$

where *E*_*mn*_ ≡ *E*_*m*_ − *E*_*n*_, *f*_*mn*_ ≡ *f*_*m*_ − *f*_*n*_ are the difference of band dispersion and Fermi–Dirac distribution, respectively, ${\Delta }_{mn}^{a}\equiv {\partial }_{{k}_{a}}{E}_{mn}/\hslash$, ${r}_{mn}^{a}\equiv i\langle m| {\partial }_{{k}_{a}}n\rangle$ is the interband transition matrix element or off-diagonal Berry connection. The finite relaxation time *τ* is considered via the Lorentzian function ${{\mathcal{L}}}_{\tau }({E}_{nm}-\omega )$.

## Supplementary information

Supplementary Information

## Data Availability

All data needed to evaluate the conclusions in the paper are present in the paper and the Supplementary Information. Additional data related to this paper could be requested from the authors.
