# Supplementary material for: Giant topological longitudinal circular photo-galvanic effect in the chiral multifold semimetal CoSi
Source: Nat Commun. 2021 Jan 8;12:154. doi: 10.1038/s41467-020-20408-5 (PMC7794406; doi:10.1038/s41467-020-20408-5)
Supplement: Supplementary file 1 — Supplementary Information [file 41467_2020_20408_MOESM1_ESM.pdf]

Supplementary Information for "Giant topological longitudinal circular photo-galvanic effect in the chiral multifold semimetal CoSi"

Zhuoliang Ni,[1] K. Wang,[2] Y. Zhang,[3,4] O. Pozo,[5] B. Xu,[6] X. Han,[1] K. Manna,[4,7] J. Paglione,[2,8] C. Felser,[4,8] A. G. Grushin,[9] F. de Juan,[10,11] E. J. Mele,[1] and Liang Wu[1,*]

[1]*Department of Physics and Astronomy, University of Pennsylvania, Philadelphia, Pennsylvania 19104, USA*

[2]*Maryland Quantum Materials Center, Department of Physics, University of Maryland, College Park, MD 20742, USA.*

[3]*Department of Physics, Massachusetts Institute of Technology, Cambridge, Massachusetts 02139, USA*

[4]*Max-Planck-Institut fur Chemische Physik fester Stoffe, 01187 Dresden, Germany*

[5]*Instituto de Ciencia de Materiales de Madrid, CSIC, Cantoblanco, Madrid, 28049, Spain*

[6]*Department of Physics and Fribourg Center for Nanomaterials, University of Fribourg, Chemin du Musée 3, CH-1700 Fribourg, Switzerland*

[7]*Present address: Department of Physics, Indian Institute of Technology Delhi, Hauz Khas, New Delhi-110016, India*

[8]*Canadian Institute for Advanced Research, Toronto, Ontario M5G 1Z8, Canada*

[9]*Univ. Grenoble Alpes, CNRS, Grenoble INP, Institut Néel, 38000 Grenoble, France*

[10]*Donostia International Physics Center, P. Manuel de Lardizabal 4, 20018 Donostia-San Sebastian, Spain*

[11]*IKERBASQUE, Basque Foundation for Science, Maria Diaz de Haro 3, 48013 Bilbao, Spain*

[*]Electronic address: liangwu@sas.upenn.edu

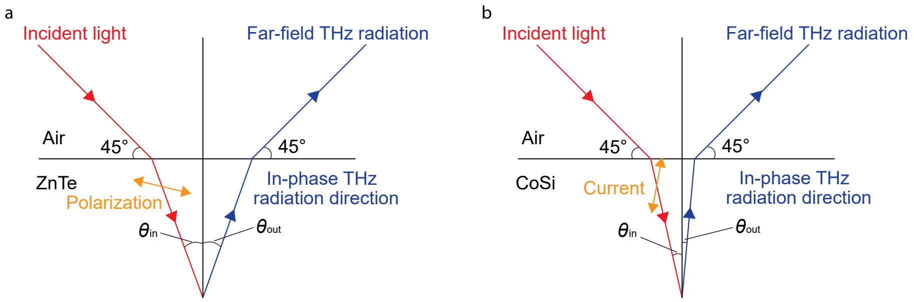

Supplementary Figure 1: **Geometry of the air/sample interface.** **a**, Geometry of the air/sample interface of ZnTe. **b**, Geometry of the air/sample interface of CoSi. Red arrows represent the direction of excitation light, and the blue arrows represent the direction of in-phase THz wave. The yellow arrows represent the polarization from the optical rectification effect in ZnTe and the current from the photogalvanic effect in CoSi. Note the current in b contains both CPGE and LPGE contribution. The CPGE contribution is strictly along the direction of the excitation light inside the sample, while the LPGE contribution is not restricted.

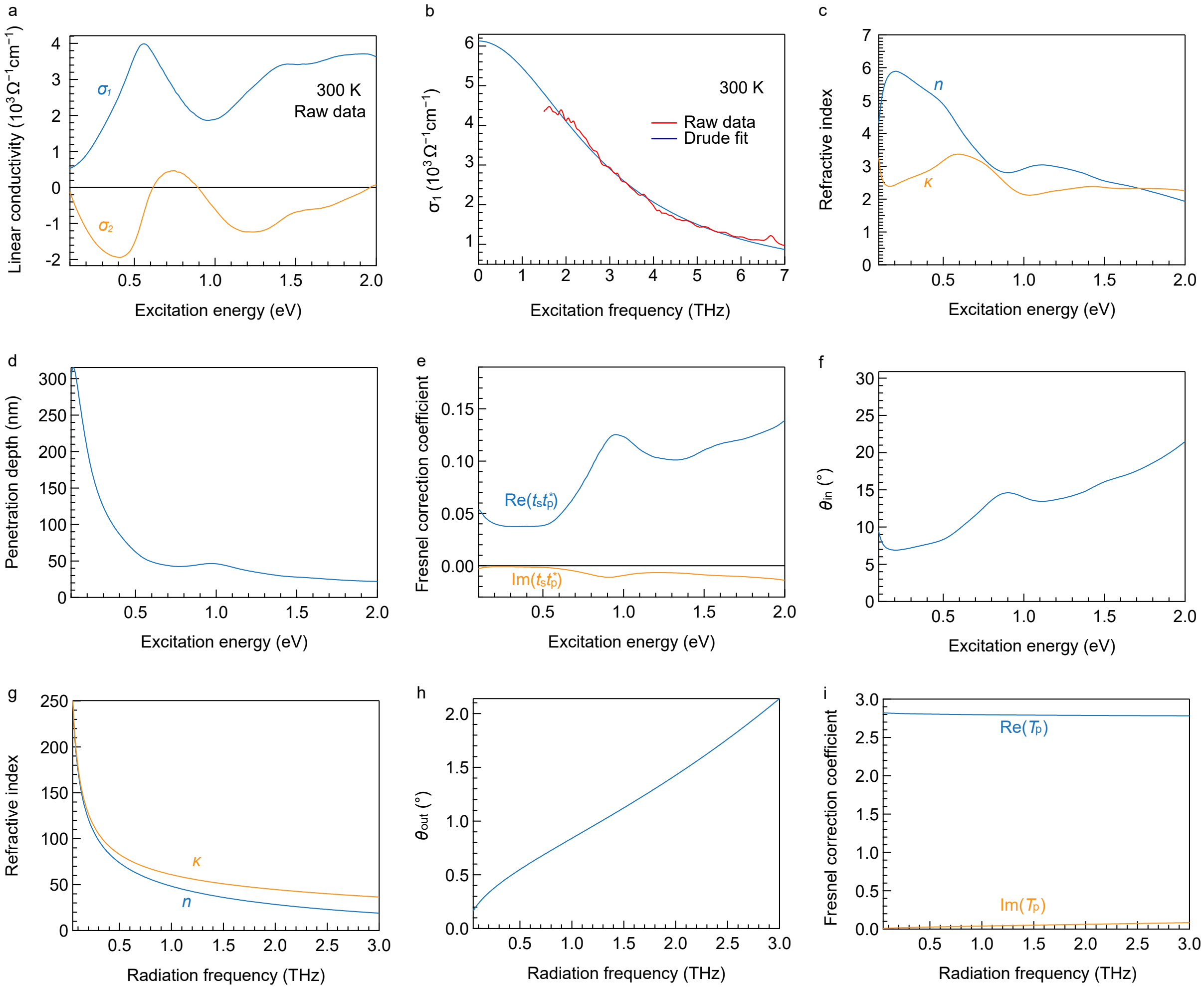

Supplementary Figure 2: **Parameters of CoSi related with the optical conductivity.** **a**, Optical conductivity of CoSi as a function of excitation energy measured at 300 K. The data is reproduced from Ref. [4]. Only 0.1-2.0 eV is shown.**b**, Drude fit (blue) of the optical conductivity raw data (red) at THz range. **c-f**, Refractive index (c), penetration depth (d), Fresnel correction coefficient (e) and θ_{in} (f) of CoSi as a function of the excitation energy. **g-i**, Refractive index (g), θ_{out} (h) and Fresnel coefficient (i) of CoSi as a function of the THz radiation frequency. These curves are derived based on the optical conductivity following Drude formula fitted in (b).

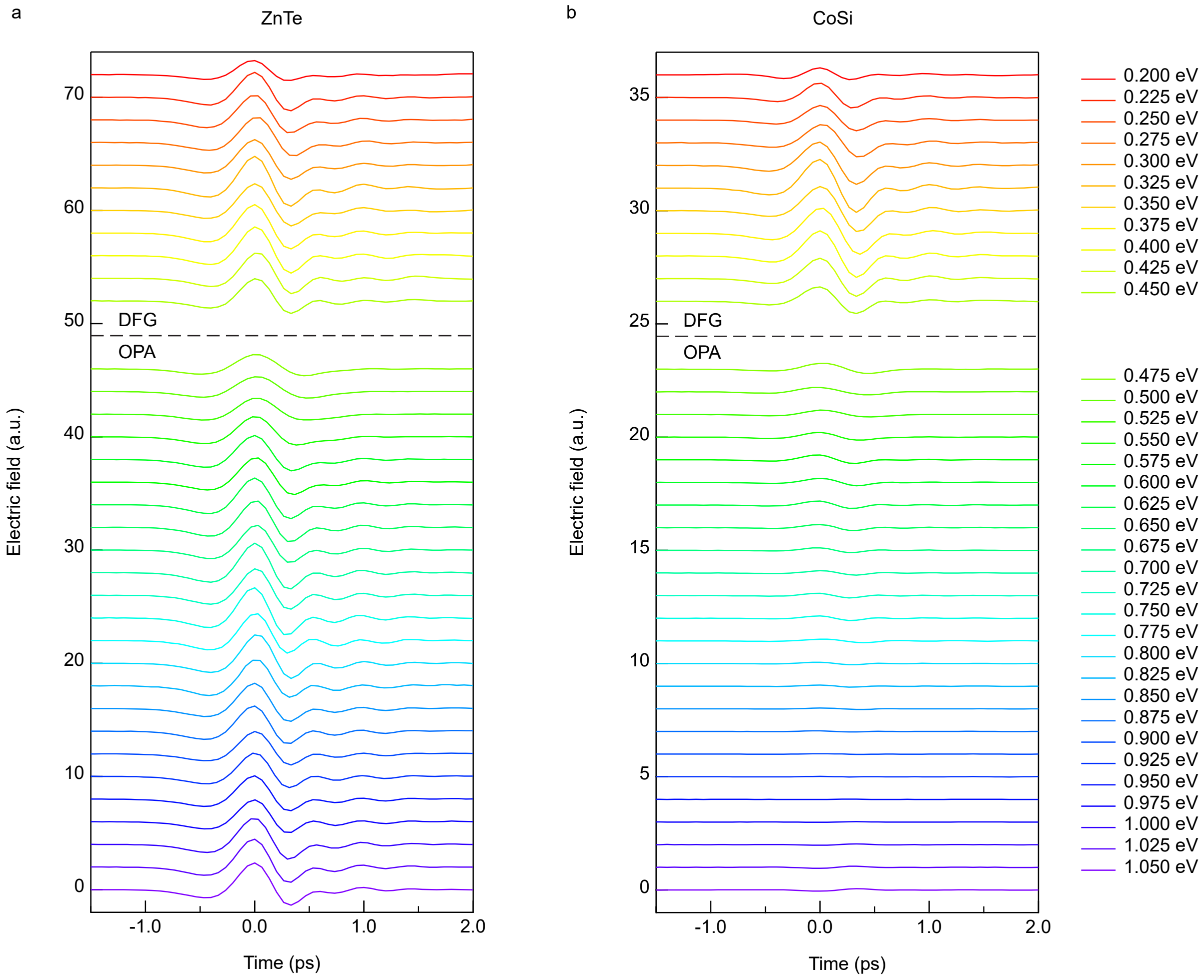

Supplementary Figure 3: **Raw data of THz time traces of optical rectification at different excitation photon energy. a**, Raw data of ZnTe. **b**, Raw data of CoSi. The data are normalized by the incident power. The CoSi CPGE data shown is one half of the difference between the in-plane THz wave of the left-handed and right-handed incident laser excitation pulses. The laser pulse energy range of 0.200-0.450 eV is generated by difference frequency generation (DFG), while the range of 0.475-1.05 eV is generated by optical parametric amplifier (OPA).

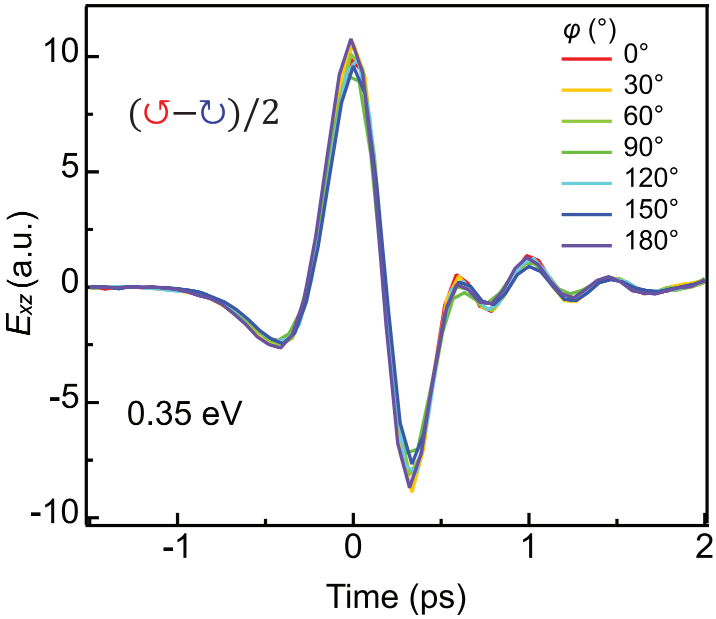

Supplementary Figure 4: **CPGE THz time traces at different sample azimuth angles ϕ at the incident photon energy of 0.35 eV.**

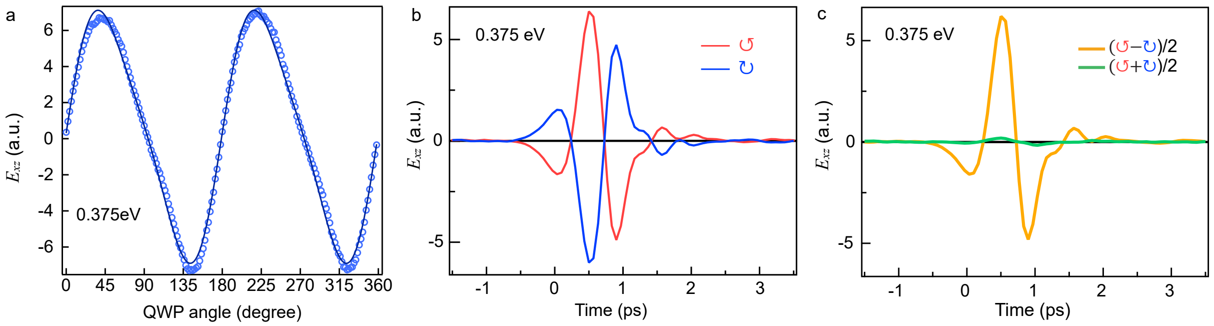

Supplementary Figure 5: **THz emission data of CoSi measured at 0.375 eV. a**, CPGE THz peak amplitude in CoSi as a function of the angle of the quarter wave plate with incident photon energy of 0.375 eV. **b**, THz wave form of the in-plane component under left-handed and right-handed incident laser pulses at 0.375 eV. **c**, CPGE and LPGE THz wave form of the in-plane component at 0.375 eV. The black line is at zero.

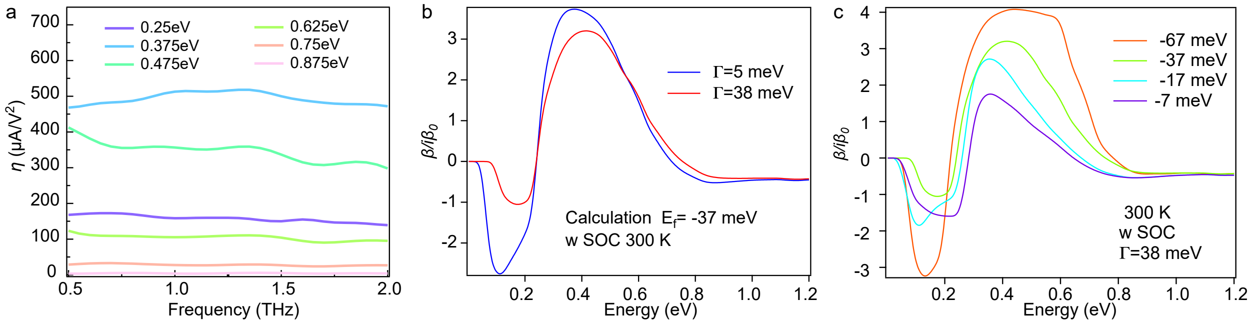

Supplementary Figure 6: **Possible CPGE quantization from the DFT. a**, CPGE current amplitude in the frequency domain at different incident photon energy in CoSi. No obvious THz frequency dependence is observed, indicating the hot-carrier time is much shorter than the laser pulse width. **b**, CPGE calculation at room temperature with SOC of two different broadening of 38 meV and 5 meV. **c**, CPGE calculation at room temperature with SOC of broadening of 38 meV with different chemical potential.

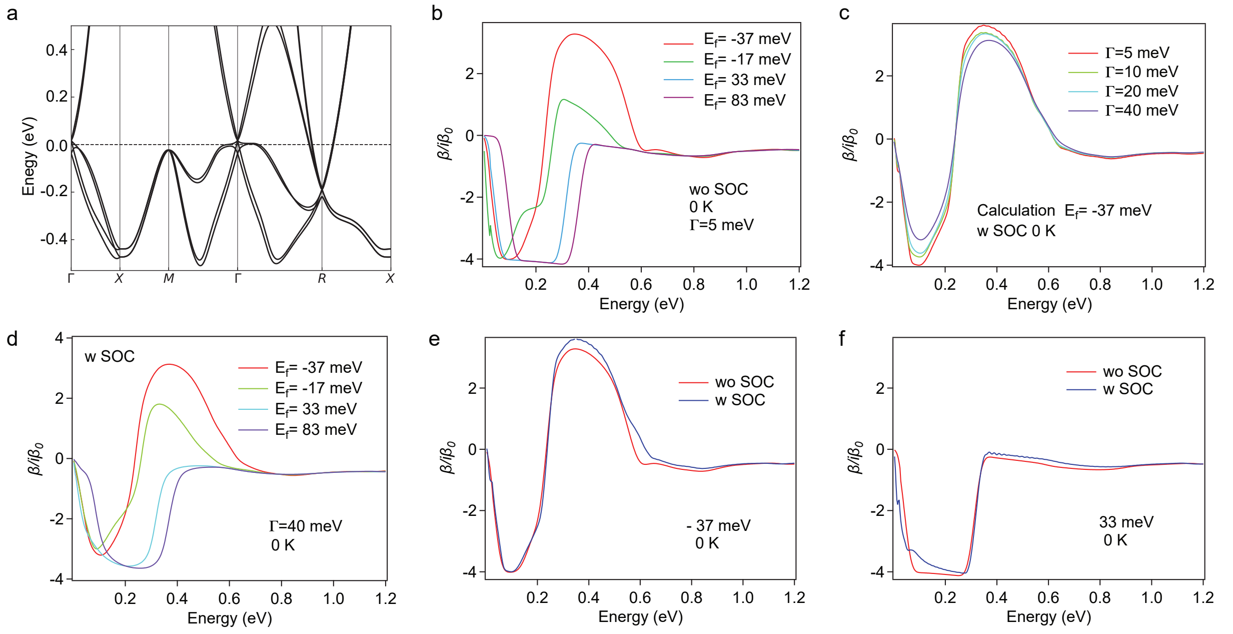

Supplementary Figure 7: **More DFT results. a**, Band structure of CoSi with spin-orbit coupling. The dashed line is at $E_f = -37$ meV. Note that the DFT gives $E_f = -20$ meV. **b**, Calculated CPGE current at T=0 K with $E_f = -37$ meV, $E_f = -17$ meV, $E_f = 33$ meV and $E_f = 83$ meV with spin-orbit coupling. **c**, Calculated CPGE current at T=0 K with $E_f = -30$ meV with spin-orbit coupling and with different broadening. **d**, Calculated CPGE current at T=0 K with a broadening of 40 meV with spin-orbit coupling and with different chemical potential. Calculated CPGE current at T=0 K with 5 meV broadening with and without spin-orbit coupling at e, $E_f = -37$ meV and f, $E_f = 33$ meV.

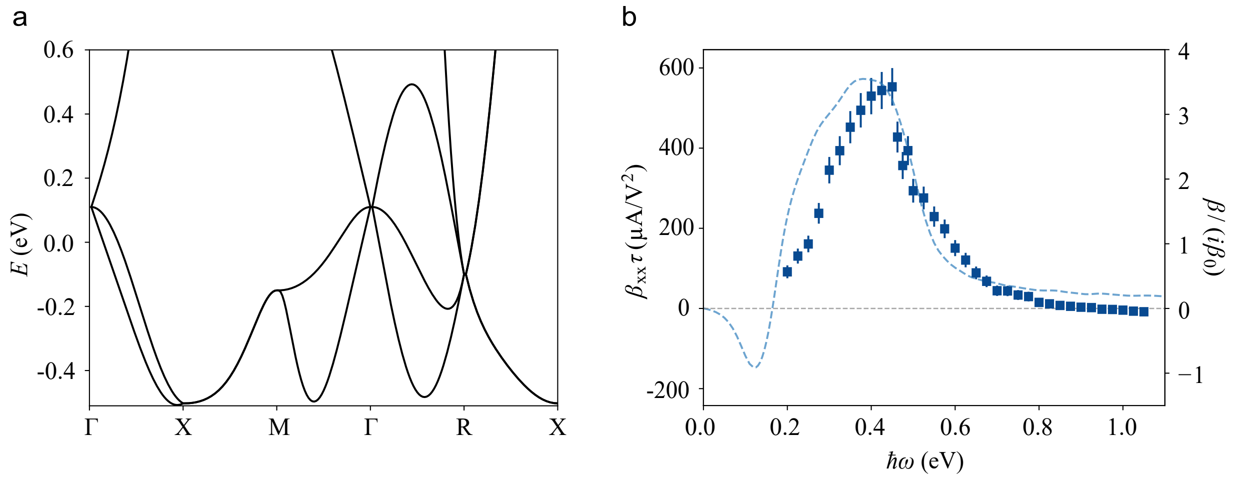

Supplementary Figure 8: **Tight-binding model calculation of the CPGE responce in CoSi. a**, Band structure obtained with the tight-binding model for space group 198 with parameters $v_1 = 1.29$, $v_p = 0.55$, and $v_2 = 0.25$ eV. $E = 0$ marks the position of the chemical potential, 110 meV below the threefold node at Γ. **b**, Corresponding CPGE obtained using Eq. (2) in the main text with a broadening of Γ=40 meV (dashed) compared to the experimental data (squares).

a

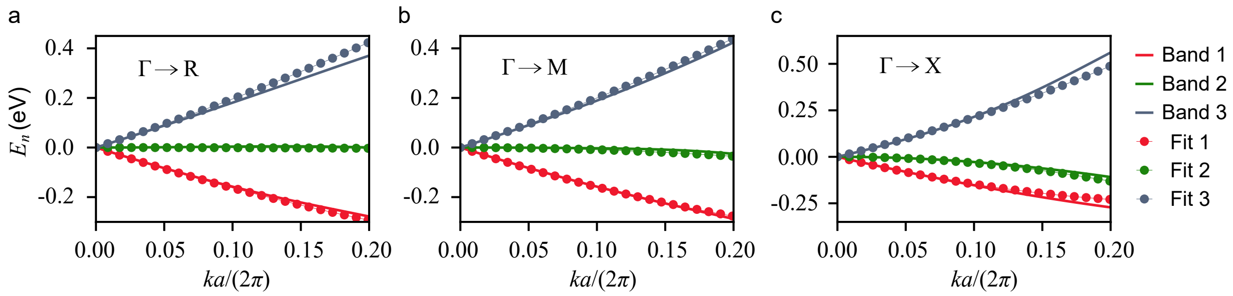

Supplementary Figure 9: **Fit of the k · p model parameters from the ab-initio energy bands. a**, Γ to R point. **b**, Γ to M point. **c**, Γ to X point. The results of fitting parameter are $(v, a, b, c) = (1.79, 1.07, -1.72, 3.26)$.

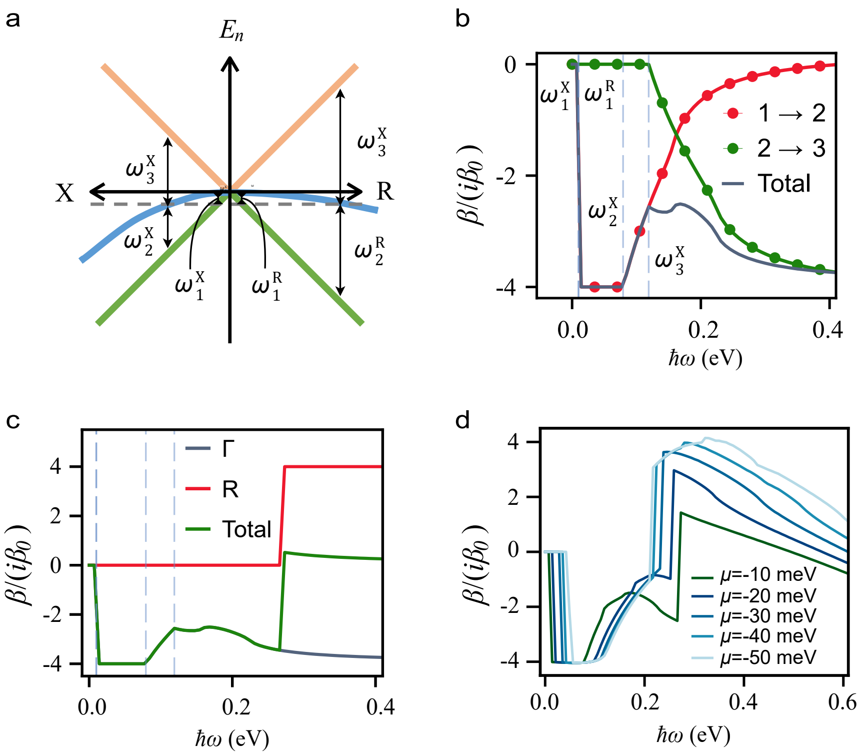

Supplementary Figure 10: **Different CPGE results from the** *ab initio* **fitting. a**, Sketch of the critical frequencies for a negative chemical potential. **b**, Γ point contribution coming from the first effect of quadratic corrections for $\mu = -10$ meV. The vertical lines show the critical frequencies corresponding to each band transition contribution. **c**, Total contribution from the Γ and R points for $\mu = -10$ meV including the effect of quadratic corrections on the energies but not on the matrix elements, showing the characteristic shape of CPGE. **d**, CPGE for different chemical potentials, now including all the effects of quadratic corrections.

Supplementary Note 1. Conversion of THz signal

As will be explained in the following part, the symmetry of CoSi only allows CPGE current along the wave vector direction inside the material determined by the incident wave vector. Normal incident laser pulses can only generate longitudinal ultrafast current, which is perpendicular to the surface and does not emit THz radiation into the free space. Therefore, in order to make THz pulses radiate out to the free space in the far-field, we choose the incident angle to be at 45 degrees in the reflection geometry. The schematic diagrams of the air-sample interface of both the benchmarking crystal ZnTe and the sample CoSi for the THz emission process are shown in Supplementary Figure 1. The incident light (red) gets refracted when entering the sample at the angle of refraction θ_{in}. The ultrafast excitation light inside the sample generates an ultrafast polarization (yellow) in ZnTe or an ultrafast current (yellow) in CoSi, both of which emit THz radiation. Note the current drawn in CoSi contains both CPGE and LPGE, so its direction is not strictly along the light vector direction. Only if CPGE is considered, the current direction is along the light vector direction due to the cubic symmetry of CoSi. In the process of THz emission, only the component of polarization or current that is perpendicular to the in-phase THz radiation direction (blue) generates the coherent THz radiation in the far-field. The direction of the in-phase THz radiation (θ_{out} off the interface normal) is determined by the refractive index at the THz range.

A. THz generation from ZnTe

A ZnTe crystal generates THz emission via optical rectification. The nonlinear polarization P under the driving electric field E is characterized by second-order susceptibility tensor $\chi^{(2)}(0;\omega,\omega)$,

$$\mathbf{P}(0) = \epsilon_0 \chi^{(2)}(0;\omega,\omega) : \mathbf{E}\ \mathbf{E}^*. \tag{S1}$$

ZnTe belongs to space group $F\bar{4}3m$, of which $\chi^{(2)}$ contains only one independent parameter,

$$\chi^{(2)} = \begin{pmatrix} \begin{pmatrix} 0 \\ 0 \\ 0 \end{pmatrix} & \begin{pmatrix} 0 \\ 0 \\ \chi_{41} \end{pmatrix} & \begin{pmatrix} 0 \\ \chi_{41} \\ 0 \end{pmatrix} \\ \begin{pmatrix} 0 \\ 0 \\ \chi_{41} \end{pmatrix} & \begin{pmatrix} 0 \\ 0 \\ 0 \end{pmatrix} & \begin{pmatrix} \chi_{41} \\ 0 \\ 0 \end{pmatrix} \\ \begin{pmatrix} 0 \\ \chi_{41} \\ 0 \end{pmatrix} & \begin{pmatrix} \chi_{41} \\ 0 \\ 0 \end{pmatrix} & \begin{pmatrix} 0 \\ 0 \\ 0 \end{pmatrix} \end{pmatrix} \tag{S2}$$

We use [1 1 0] cut ZnTe as a THz emission reference. To simplify the following calculation, we rotate $\chi^{(2)}$ to align the [1 1 0] direction of crystal along the z axis in the lab frame,

$$\chi^{(2)} = \begin{pmatrix} \begin{pmatrix} \frac{3\chi_{41}(\sin(2\phi)+1)(\cos(\phi)-\sin(\phi))}{2\sqrt{2}} \\ \frac{\chi_{41}(3\sin(2\phi)-1)(\sin(\phi)+\cos(\phi))}{2\sqrt{2}} \\ 0 \end{pmatrix} & \begin{pmatrix} \frac{\chi_{41}(3\sin(2\phi)-1)(\sin(\phi)+\cos(\phi))}{2\sqrt{2}} \\ -\frac{\chi_{41}(3\sin(2\phi)+1)(\cos(\phi)-\sin(\phi))}{2\sqrt{2}} \\ 0 \end{pmatrix} & \begin{pmatrix} 0 \\ 0 \\ \frac{\chi_{41}(\sin(\phi)-\cos(\phi))}{\sqrt{2}} \end{pmatrix} \\ \begin{pmatrix} \frac{\chi_{41}(3\sin(2\phi)-1)(\sin(\phi)+\cos(\phi))}{2\sqrt{2}} \\ -\frac{\chi_{41}(3\sin(2\phi)+1)(\cos(\phi)-\sin(\phi))}{2\sqrt{2}} \\ 0 \end{pmatrix} & \begin{pmatrix} -\frac{\chi_{41}(3\sin(2\phi)+1)(\cos(\phi)-\sin(\phi))}{2\sqrt{2}} \\ \frac{3\chi_{41}(\sin(\phi)+\cos(\phi))(1-2\sin(\phi)\cos(\phi))}{2\sqrt{2}} \\ 0 \end{pmatrix} & \begin{pmatrix} 0 \\ 0 \\ -\frac{\chi_{41}(\sin(\phi)+\cos(\phi))}{\sqrt{2}} \end{pmatrix} \\ \begin{pmatrix} 0 \\ 0 \\ \frac{\chi_{41}(\sin(\phi)-\cos(\phi))}{\sqrt{2}} \end{pmatrix} & \begin{pmatrix} 0 \\ 0 \\ -\frac{\chi_{41}(\sin(\phi)+\cos(\phi))}{\sqrt{2}} \end{pmatrix} & \begin{pmatrix} \frac{\chi_{41}(\sin(\phi)-\cos(\phi))}{\sqrt{2}} \\ -\frac{\chi_{41}(\sin(\phi)+\cos(\phi))}{\sqrt{2}} \\ 0 \end{pmatrix} \end{pmatrix}, \tag{S3}$$

where we have a free parameter ϕ since the crystal can rotate freely along the z-axis and ϕ is the angle between crystal direction [1 -1 0] and the x-axis in the lab.

For all the experiments in this paper, we make $\phi = 0$, align the polarization of incident light along the x-axis, and detect THz wave component in the xz-plane, so

$$\chi^{(2)} = \begin{pmatrix} \begin{pmatrix} \frac{3\chi_{41}}{2\sqrt{2}} \\ -\frac{\chi_{41}}{2\sqrt{2}} \\ 0 \end{pmatrix} & \begin{pmatrix} -\frac{\chi_{41}}{2\sqrt{2}} \\ -\frac{\chi_{41}}{2\sqrt{2}} \\ 0 \end{pmatrix} & \begin{pmatrix} 0 \\ 0 \\ -\frac{\chi_{41}}{\sqrt{2}} \end{pmatrix} \\ \begin{pmatrix} -\frac{\chi_{41}}{2\sqrt{2}} \\ -\frac{\chi_{41}}{2\sqrt{2}} \\ 0 \end{pmatrix} & \begin{pmatrix} -\frac{\chi_{41}}{2\sqrt{2}} \\ \frac{3\chi_{41}}{2\sqrt{2}} \\ 0 \end{pmatrix} & \begin{pmatrix} 0 \\ 0 \\ -\frac{\chi_{41}}{\sqrt{2}} \end{pmatrix} \\ \begin{pmatrix} 0 \\ 0 \\ -\frac{\chi_{41}}{\sqrt{2}} \end{pmatrix} & \begin{pmatrix} 0 \\ 0 \\ -\frac{\chi_{41}}{\sqrt{2}} \end{pmatrix} & \begin{pmatrix} -\frac{\chi_{41}}{\sqrt{2}} \\ -\frac{\chi_{41}}{\sqrt{2}} \\ 0 \end{pmatrix} \end{pmatrix}, \tag{S4}$$

$$\mathbf{E} = E_0 \begin{pmatrix} t_p \cos(\theta_{in}) \\ 0 \\ t_p \sin(\theta_{in}) \end{pmatrix}, \tag{S5}$$

$$P_{eff} = \mathbf{P} \cdot \begin{pmatrix} \cos(\theta_{out}) \\ 0 \\ \sin(\theta_{out}) \end{pmatrix}, \tag{S6}$$

where t_p is the Fresnel coefficient, θ_{in} is the incident angle inside the sample, θ_{out} is the refracted angle of the THz light inside the sample (See Supplementary Figure 1), and P_{eff} is the component of $\mathbf{P}$ perpendicular to the detecting direction. Combing (S1)(S4)(S5)(S6), we get

$$P_{eff} = \frac{4\sin(2\theta_{in})\sin(\theta_{out}) + 5\cos 2(\theta_{in})\cos(\theta_{out}) + \cos(\theta_{out})}{4\sqrt{2}} t_p^2 \epsilon_0 \chi_{41} E_0^2 \equiv \gamma^{ZnTe} \epsilon_0 \chi_{41} E_0^2. \tag{S7}$$

Here, P_{eff} is in the time domain and has a pulsed shape with a time scale of picosecond, so its Fourier transform $P_{eff}(\Omega)$ is in the THz range. The generated THz field strength just outside the sample surface in the frequency domain can be written as

$$E_{eff}^{ZnTe}(\Omega) = \frac{Z_0 \Omega P_{eff}(\Omega)}{2n^{ZnTe}(\Omega)} T_p^{ZnTe} l^{ZnTe}(\Omega), \tag{S8}$$

where Ω is THz frequency, Z_0 is the impedance of free space, $P_{eff}(\Omega)$ is the Fourier transform of P_{eff}, $n^{ZnTe}(\Omega)$ is the refractive index of ZnTe for frequency Ω, T_p^{ZnTe} is the Fresnel coefficient, and l^{ZnTe} is the coherent length of the THz wave in ZnTe.

B. THz generation from CoSi

CoSi belongs to the $P2_13$ space group, and its second-order conductivity tensor has the same form as (S2) except that the parameter is a complex number $\sigma + i\eta$. In the experiment, samples with (111) surface are measured. After a rotation transformation of the conductivity tensor, the total current $\mathbf{j}$ can be written as

$$\mathbf{j} = \sigma^{(2)} : \mathbf{E}\,\mathbf{E}^* \tag{S9}$$

with

$$\sigma^{(2)} = \begin{pmatrix} \begin{pmatrix} \sqrt{\frac{2}{3}}\sigma\cos(3\phi) \\ \sqrt{\frac{2}{3}}\sigma\sin(3\phi) \\ -\frac{\sigma}{\sqrt{3}} \end{pmatrix} & \begin{pmatrix} \sqrt{\frac{2}{3}}\sigma\sin(3\phi) \\ -\sqrt{\frac{2}{3}}\sigma\cos(3\phi) \\ i\eta \end{pmatrix} & \begin{pmatrix} -\frac{\sigma}{\sqrt{3}} \\ -i\eta \\ 0 \end{pmatrix} \\ \begin{pmatrix} \sqrt{\frac{2}{3}}\sigma\sin(3\phi) \\ -\sqrt{\frac{2}{3}}\sigma\cos(3\phi) \\ -i\eta \end{pmatrix} & \begin{pmatrix} -\sqrt{\frac{2}{3}}\sigma\cos(3\phi) \\ -\sqrt{\frac{2}{3}}\sigma\sin(3\phi) \\ -\frac{\sigma}{\sqrt{3}} \end{pmatrix} & \begin{pmatrix} i\eta \\ -\frac{\sigma}{\sqrt{3}} \\ 0 \end{pmatrix} \\ \begin{pmatrix} -\frac{\sigma}{\sqrt{3}} \\ i\eta \\ 0 \end{pmatrix} & \begin{pmatrix} -i\eta \\ -\frac{\sigma}{\sqrt{3}} \\ 0 \end{pmatrix} & \begin{pmatrix} 0 \\ 0 \\ \frac{2\sigma}{\sqrt{3}} \end{pmatrix} \end{pmatrix}, \tag{S10}$$

where the operators σ and η describe LPGE and CPGE contributions, respectively, and ϕ is the angle between the sample axis [2 -1 -1] and x axis in the lab frame. For most of our experiments, we use only one QWP to change the polarization of the incident light and fix ϕ=90 degrees. The electric field after the QWP is

$$\mathbf{E} = E_0 \begin{pmatrix} t_p\cos(\theta_{in})\left(\cos^2(\theta) + i\sin^2(\theta)\right) \\ (1-i)t_s\sin(\theta)\cos(\theta) \\ t_p\sin(\theta_{in})\left(\cos(\theta) + i\sin^2(\theta)\right) \end{pmatrix} \tag{S11}$$

With S9 and the geometry shown in Supplementary Figure 1, one can calculate the current component coupled to the free space THz radiation in the far field,

$$\begin{aligned} j^{xz}_{eff}(\theta) =& i\eta E_0^2\sin(2\theta)\sin\left(\theta_{\text{in}}+\theta_{\text{out}}\right)\left(\text{Re}(t_st_p^*) + \text{Im}(t_st_p^*)\cos(2\theta)\right) \\ &- \frac{\sigma E_0^2}{8\sqrt{3}}(\cos(4\theta)+3)\left|t_p\right|^2\left(2\sin\left(2\theta_{\text{in}}\right)\cos\left(\theta_{\text{out}}\right) + \left(1-3\cos\left(2\theta_{\text{in}}\right)\right)\sin\left(\theta_{\text{out}}\right)\right) \\ &- \frac{\sigma E_0^2}{2\sqrt{3}}\sin(2\theta)\left(\sin(2\theta)\left|t_s\right|^2\sin\left(\theta_{\text{out}}\right) + \sqrt{2}\cos\left(\theta_{\text{in}}\right)\cos\left(\theta_{\text{out}}\right)\left((\cos(2\theta)+i)t_pt_s^* + (\cos(2\theta)-i)t_st_p^*\right)\right) \end{aligned} \tag{S12}$$

The parameters $t_s t_p^*$, θ_{in} and θ_{out} used in this equation can be determined by the linear optical conductivity of CoSi, which is shown in the main text Supplementary Figure 3c. In Supplementary Figure 2 we show $t_s t_p^*$ (Supplementary Figure 2e) and θ_{in}Supplementary Figure 2f) as a funcition of excitation energy and θ_{out} Supplementary Figure 2h) as a function of radiation frequency (See details in the Supplementary Note 1 E).

Considering $\mathrm{Re}(t_s t_p^*) \gg \mathrm{Im}(t_s t_p^*)$ and $\theta_{out} \ll 1$, S12 can be further simplified to

$$\begin{aligned} j_{eff}^{xz}(\theta) =& \eta E_0^2 \sin(2\theta) \sin(\theta_{\mathrm{in}} + \theta_{\mathrm{out}}) \, \mathrm{Re}(t_s t_p^*) \\ & - \frac{\sigma E_0^2}{4\sqrt{3}} (\cos(4\theta) + 3) \, |t_p|^2 \sin(2\theta_{\mathrm{in}}) \cos(\theta_{\mathrm{out}}) \\ & - \frac{\sigma E_0^2}{\sqrt{6}} \sin(4\theta) \cos(\theta_{\mathrm{in}}) \, \mathrm{Re}(t_s t_p^*). \end{aligned} \tag{S13}$$

To get pure CPGE, we measure the current difference between θ=45 degrees and θ=135 degrees,

$$j_{eff}^{CPGE} = \frac{1}{2}\left(j_{eff}^{xz}(\frac{3\pi}{4}) - j_{eff}^{xz}(\frac{\pi}{4}) \right) = \eta \, \mathrm{Re}\,(t_s t_p) \sin(\theta_{\mathrm{in}} + \theta_{\mathrm{out}}) \, E_0^2 = \gamma^{CoSi} \eta E_0^2. \tag{S14}$$

Note the above equation is true for any crystal orientation, i.e., any ϕ and surface index.

We then calculate the generated current out of the incident plane, with the same approximation $\mathrm{Re}(t_s t_p^*) \gg \mathrm{Im}(t_s t_p^*)$ and $\theta_{out} \ll 1$,

$$j_{eff}^{y}(\theta) = -\frac{\sigma E_0^2}{4\sqrt{3}} \left(\sqrt{2}\,|tp|^2 (\cos(4\theta) + 3) \cos^2(\theta_{in}) + 2 t_s t_p^* \sin(4\theta) \sin(\theta_{in}) + \sqrt{2}\,|t_s|^2 (\cos(4\theta) - 1) \right). \tag{S15}$$

Note this result does not have any term with η, meaning CPGE does not produce out-of-plane photocurrent, which is consistent with the longitudinal response. In the main text, we write: $E_{xz}(\theta) = A\sin(2\theta) + B_2 \sin(4\theta) + C_2 \cos(4\theta) + D_2$ and $E_y(\theta) = B_1 \sin(4\theta) + C_1 \cos(4\theta) + D_1$. Here E_{xz} is related with j_{eff}^{xz} and E_y is related with j_{eff}^{y}, and one can see that coefficients A,B_1,B_2,C_1,C_2,D_1,D_2 are determined by the CPGE conductivity η and LPGE conductivity σ.

Similar to ZnTe, the generated THz field of CoSi in the near field is

$$E_{eff}^{CoSi}(\Omega) = \frac{Z_0 j_{eff}(\Omega)}{2n^{CoSi}(\Omega)} T_p^{CoSi} l^{CoSi}(\Omega), \tag{S16}$$

where $j_{eff}(\Omega)$ is the Fourier transform of j_{eff}, $n^{CoSi}(\Omega)$ is the refractive index of CoSi, T_p^{CoSi} is the Fresnel coefficient, and l^{CoSi} is the penetration depth of the incident light in CoSi. Note $n^{CoSi}(\Omega)$, T_p^{CoSi} and l^{CoSi} are determined by the optical conductivity of CoSi and their dependence on radiation frequency or excitation energy are shown in Supplementary Figure 2 (See details in the Supplementary Note 1 E).

C. Derivation of CoSi response by the benchmarking ZnTe

The emitted THz wave is collected by two off-axis parabolic mirrors and focused on a ZnTe detector. A probe beam with 35 fs pulses is also focused on the ZnTe detector. The electric-optical sampling signal in frequency domain is

$$\frac{\Delta I(\Omega)}{I} = \frac{\omega n^3 r_{41} L t_p}{2c} E_{THz}(\Omega). \tag{S17}$$

Since ZnTe and CoSi are measured in the same setup, they should have the same collection efficiency for a specific THz frequency, thus

$$\frac{(\frac{\Delta I(\Omega)}{I})^{CoSi}}{(\frac{\Delta I(\Omega)}{I})^{ZnTe}} = \frac{E_{eff}^{CoSi}(\Omega)}{E_{eff}^{ZnTe}(\Omega)}. \tag{S18}$$

Combining the equations above,

$$\eta(\Omega) = \frac{(\frac{\Delta I(\Omega)}{I})^{CoSi}}{(\frac{\Delta I(\Omega)}{I})^{ZnTe}} \frac{n^{CoSi}(\Omega)}{n^{ZnTe}(\Omega)} \frac{T_p^{ZnTe}}{T_p^{CoSi}} \frac{l^{ZnTe}}{l^{CoSi}} \frac{\gamma^{ZnTe}}{\gamma^{CoSi}} \Omega \epsilon_0 \chi_{41} \tag{S19}$$

D. Derivation of effective emission depth

Incident ultrafast light pulses consist of a distribution of frequency around the center photon energy. Generating current in CoSi (and polarization in the case of ZnTe) in the THz frequency involves with difference frequency generation (DFG) within the pulse frequency.

When two electric fields (E_a and E_b) with frequency $\omega_a = \omega + \Omega/2$ and $\omega_b = \omega - \Omega/2$ are incident on the sample surface, the electric fields inside the sample at a depth of d is $E_a = E_{a0} e^{ik_a d/\cos(\theta_{in}) - i(\omega+\Omega/2)t}$ and $E_b = E_{b0} e^{ik_b d/\cos(\theta_{in}) - i(\omega-\Omega/2)t}$. The generated current with THz frequency Ω at the depth of d is

$$j_\Omega(t) = \sigma(\Omega) E_{a0} E_{b0} e^{i(k_a - k_b^*)d/\cos(\theta_{in})} e^{-i\Omega t}. \tag{S20}$$

The THz wave generated by the current in depth d on the detector is

$$E_\Omega(t) = \Gamma(\Omega)\sigma(\Omega) E_{a0} E_{b0} e^{i(k_a - k_b^*)d/\cos(\theta_{in})} e^{-ik_\Omega d/\cos(\theta_{out}) - i\Omega t}, \tag{S21}$$

where $\Gamma(\Omega)$ is the conversion efficiency from THz current in the sample to THz wave on the detector at frequency Ω. After integrating over propagation direction of output THz wave, one gets

$$E_\Omega(t) = \frac{\Gamma(\Omega)\sigma(\Omega) E_{a0} E_{b0} e^{i(k_a - k_b^*)e^{-i\Omega t}}}{i(k_a - k_b^*) d \frac{\cos\theta_{in}}{\cos\theta_{out}} - ik_\Omega d} \tag{S22}$$

So the effective emission depth is

$$l_\Omega = \left| \frac{1}{i(k_a - k_b^*)d\frac{\cos\theta_{in}}{\cos\theta_{out}} - ik_\Omega d} \right| \tag{S23}$$

In the case of ZnTe, one can assume k_a and k_b to be purely real so

$$l^{ZnTe}(\Omega) = \frac{c}{(n_g(\omega)\frac{\cos(\theta_{out})}{\cos(\theta_{in})} + n^{ZnTe}(\Omega))\Omega}, \tag{S24}$$

where $n_g(\omega)$ is the group index of ZnTe at the frequency ω.

In the case of CoSi, k_a and k_b have the imaginary part $\mathrm{Im}(n^{CoSi}(\omega))\omega/c$ and is much larger than $(n_g + k_\Omega)\Omega/c$, so

$$l^{CoSi}(\Omega) = \frac{c}{2\,\mathrm{Im}(n^{CoSi}(\omega))\frac{\cos(\theta_{out})}{\cos(\theta_{in})}\omega}. \tag{S25}$$

The excitation energy dependence of $l^{CoSi}(\Omega)$ is shown in Supplementary Figure 2d. Note in the figure we ignore its dependence on θ_{out} by the approxiamtion $\theta_{out} \ll 1$. See details in Supplementary Note 1 E.

E. Parameters based on optical conductivity in CoSi

During the above conversion process, many parameters relied on the optical conductivity in both THz and excitation energy range are needed. Our linear conductivity measurement in CoSi is summarized and discussed detailly in Ref. [4]. In this paper, we reproduce the data measured at room temperature at both the excitation energy range (Supplementary Figure 2a) and the THz radiation range (Supplementary Figure 2b). For excitation energy range, we calculate the refractive index Supplementary Figure 2c), penetration depth (Supplementary Figure 2d), Fresnel correction coefficient (Supplementary Figure 2e) and refracted angle θ_{in} (Supplementary Figure 2f) as a function of excitation energy. As for the THz range, the lowest energy we get from the linear conductivity measurement is 1.6 THz, while the THz wave we detect in the THz emission experiment is 0.2-2 THz. To get the linear response below 1.6 THz, we fit the measured data from 1.6-6.0 THz by Drude formula. The zero-frequency extrapolation agrees with the DC transport conductivity. The best fit is shown in Supplementary Figure 2a with the broadening $\Gamma = \hbar/\tau$=14 meV and transport conductivity $\sigma_0 = 6.1 \times 10^3\ \Omega^{-1}\mathrm{cm}^{-1}$. We then derive the refractive index (Supplementary Figure 2g), the angle of in-phase radiation direction θ_{out} Supplementary Figure 2h) and the Fresnel coefficient (Supplementary Figure 2i) in the THz range.

F. THz time traces of optical rectification in ZnTe and CPGE in CoSi at different excitation photon energy

Here we show the raw data of THz waveform in the time domain of optical rectification in ZnTe and CPGE contribution of CoSi at 0.200 -1.050 eV in Supplementary Figure 3. All the data are normalized by the incident power. ZnTe is excited by horizontally polarized (xz-plane) incident laser pulses, and the in-plane component THz wave is collected. The CoSi CPGE data shown is one half of the difference between the in-plane THz wave of the left-handed and right-handed incident laser excitation pulses. The laser pulse energy range of 0.200-0.450 eV is generated by difference frequency generation (DFG), while the range of 0.475-1.05 eV is generated by optical parametric amplifier (OPA). In the DFG range, a germanium focusing lens and a MgF_2 achromatic quarter-wave plate are used, and in the OPA regime, a BaF_2 focusing lens and a quartz-MgF_2 achromatic quarter-wave plate are used. (See more details in Methods). A broadening of the waveform and a decreasing of the peak signal are observed at the low-energy edge of both DFG and OPA, which is caused by a larger laser pulse width from the total dispersion of the quarter-wave plate, the focusing lens and the linear polarizer. In general, the ZnTe shows an energy-independent signal as expected, which serves as a good benchmarking material. For the CoSi CPGE signal, the raw signal increases dramatically from 1.050 eV and peaks at around 0.400 eV.

Supplementary Note 2. Additional symmetry-related measurement

A. CPGE signal at different sample azimuth angles at 0.35 eV

Besides the measurement performed at 0.50 eV shown in the main text Figure 2g, CPGE signals at different sample azimuth angles are also measured at 0.35 eV, which is close to the CPGE peak. The measured THz time traces in different azimuth angles are shown in Supplementary Figure 4. All of the curves overlap well, which is consistent with the symmetry analysis.

B. THz peak signal at 0.375 eV as a function of quarter-wave plate angle

The measured E_{xz} as a function of the quarter-wave plate angle is shown in Supplementary Figure5a. The in-plane THz wave form under left-handed and right-handed laser pulses are shown in Supplementary Figure 5b. The two curves are almost identical to each other with the opposite sign. The extracted CPGE and LPGE components are shown in Supplementary Figure 5c. From

these two plots, one can see that CPGE much larger than LPGE at 0.375 eV. The black curve in Supplementary Figure 5 is the fit by $E_{xz}(\theta) = A\sin(2\theta) + B_2\sin(4\theta) + C_2\cos(4\theta) + D_2$. Note that $E_y(\theta) = B_1\sin(4\theta) + C_1\cos(4\theta) + D_1$. At 0.5 eV, fitting E_{xz} and E_y leads to $A_2 : B_2 : C_2 : D_2 = 155.25 : 78.09 : 7.42 : 20.84$ and $A_1 : B_1 : C_1 : D_1 = 0.51 : -5.86 : 77.33 : 117.62$. At 0.375 eV, fitting E_{xz} gives rise to $A_2 : B_2 : C_2 : D_2 = 380.73 : 38.32 : 2.98 : 8.37$.

Supplementary Note 3. Comparison with Quantized CPGE

The CPGE current is also called the injection current, of which the generation rate is proportional to incident power. The predicted quantized CPGE current satisfies

$$\frac{\partial j}{\partial t} = \beta_{xx} E_0^2(t). \tag{S26}$$

where $\beta_{xx} = i\beta_0/3 = i\frac{\pi e^3}{3h^2}$ in the quantization regime. If we assume the hot carrier lifetime in the sample to be τ, then the Drude model for the current in the frequency domain is

$$i\Omega j(\Omega) = \beta_{xx}\mathcal{F}(E_0^2(t)) - \frac{j(\Omega)}{\tau} \tag{S27}$$

which yields

$$j(\Omega) = \frac{\beta_{xx}}{i\Omega + 1/\tau}\mathcal{F}(E_0^2(t)) \equiv \eta\mathcal{F}(E_0^2(t)), \tag{S28}$$

where $\mathcal{F}(E_0^2(t))$ is the Fourier transform of $E_0^2(t)$, and where we have defined $\eta = \frac{\beta_{xx}}{i\Omega+1/\tau}$. As shown in Supplementary Figure 6a, we did not observe an obvious THz frequency dependency of η, which is consistent with the fact that τ is much shorter than the laser pulse width, justifying the approximation $\eta \approx \beta_{xx}\tau$.

Since the measured peak of β_{xx} is 1.1C, in units of the quantization constant $\beta_0 = \frac{\pi e^3}{h^2}$, and the measured $\beta\tau$ is around $550\mu A/V^2$, we have

$$\beta\tau = \beta\frac{\hbar}{\Gamma} = 1.1\frac{\pi e^3}{h^2}\frac{\hbar}{\Gamma} \tag{S29}$$

$$= 1.1\frac{\pi e^3}{4\pi^2\hbar eV}\frac{eV}{\Gamma} \tag{S30}$$

$$= 21\frac{\mu A}{V^2}\frac{eV}{\Gamma}, \tag{S31}$$

Therefore, we determine the broadening factor Γ to be 38 meV, which corresponds to a relaxation time of $\tau = \frac{\hbar}{\Gamma} = 17fs$. As shown in Supplementary Figure 6b, when the chemical potential is at -37 meV, calculations with broadening of 5 meV and 38 meV could reproduce the profile

(width) of experimental data, but apparently, the calculation of 5 meV broadening would give rise to a peak which is 7-8 times too high considering the extended relaxation time. Therefore, as stated in the main text, the broadening of 38 meV is chosen to constrain the width and the peak of the measured CPGE spectrum. In general, the hot-electron lifetime, and hence Γ, is energy-dependent. However, as shown in Supplementary Figure 3a in the main text, the assumption of an energy-independent broadening of 38 meV results in a calculated CPGE that matches well the experimental data between 0.25 eV and 0.7 eV. Calculations of different chemical potential with a 38 meV broadening are shown in Supplementary Figure 6c. The curve with $E_f = -37$ meV matches the photon-energy dependence well, and therefore we interpret this value as the Fermi level of our CoSi sample.

Supplementary Note 4. Possible CPGE quantization from the DFT

Supplementary Figure 7a shows the band structure of CoSi with spin-orbit coupling (SOC). The threefold nodes split into a four-fold spin 3/2 node and a Weyl node with ∼20 meV separation, much smaller than the 114 meV splitting in RhSi, which indicates that SOC is relatively small in CoSi. Consequently, we use 20 meV as an estimate for the energy scale of SOC in this compound. Supplementary Figure 7b shows the CPGE current calculation at 0 K with different chemical potential with a broadening of 5 meV without SOC. It shows a wide quantization plateau from the Γ point when the Fermi level is above the threefold node, other than the narrow quantization region at $E_f = -37$ meV as shown in the main text.

We further study the effect of changing the hot electron lifetime τ on the CPGE spectrum.Supplementary Figure 7c shows the CPGE current calculation at 0 K with the chemical potential of −37 meV but with different broadening factors $\hbar/\tau$. The quantized plateau at ∼ 100 meV disappears when the broadening reaches 10 meV. Moreover, Supplementary Figure 7d shows that with the broadening of 40 meV, the dip around 0.2 eV is not quantized even when the chemical potential is above the multifold nodes at Γ. Therefore, longer hot-electron lifetime or electron doping is essential for the observation of quantized CPGE in CoSi. Another possibility discussed in the main text is the hole doping to $E_f = -67$ meV, which turns on the transitions for the multifold nodes at the R point. The resulting wide quantization plateau around 0.4 eV is quite robust even up to room temperature. Its detection would not require a low-frequency and low-temperature measurement.

Since large SOC would remove the CPGE quantization, we also compared the results

with/without SOC. Supplementary Figure 7e-f shows the calculation of CPGE current at two different chemical potentials with and without SOC. One can see that the difference is quite small, especially in the experimental measurement regime of 0.2 eV - 1.1 eV, which proves CoSi is an ideal platform for the realization of quantized CPGE. The small differences between calculations performed with and without SOC support the validity of the spinless $\mathbf{k}\cdot\mathbf{p}$ model used in the main text, and the four-band tight-binding model in Supplementary Note 5 used to reproduce the dip-peak structure of the ab-initio CPGE spectrum.

Supplementary Note 5. CPGE from a tight-binding model for CoSi

In this note we calculate the CPGE using a tight-binding model that captures all symmetries of space group 198. This model was previously used to study RhSi in the same space group [1–3], and CoSi [4]. Neglecting spin-orbit coupling (see Supplementary Note. 4), the tight-binding model is defined by three material-dependent parameters, v_1, v_p, and v_2, which we take as $v_1 = 1.29$, $v_p = 0.55$, and $v_2 = 0.25$ eV for CoSi [4]. To this model we add a constant energy shift of 0.65 eV, which amounts to choosing the chemical potential to be 110 meV below the node at Γ. This chemical potential is chosen to reach the best agreement with the experimental data. The resulting bands are shown in Supplementary Figure 8a. Compared to DFT these bands capture well the linearly dispersing multifold bands and the separation of the multifold nodes in energy and momentum space. However, this model overestimates the bandwidth of the multifold bands, and the quadratic corrections to the flat band at Γ compared to DFT.

The calculation of the CPGE follows Ref. [2] and uses Eq. (2) in the main text. To incorporate the effect of disorder, we add a phenomenological broadening of the Dirac delta function $\delta(x) \to \frac{1}{\pi}\frac{\Gamma}{x^2+\Gamma^2}$, where $\Gamma = \hbar/\tau$. To compare with experiment, we plot $\beta_{xx}\tau$ as discussed in the Methods and Supplementary Note 3. The result with $\Gamma = 40$ meV ($\tau \approx 16$ fs) and $T = 0$ is shown in Supplementary Figure 8b. The curve shows the peak-dip feature discussed in the main text and in Supplementary Note 6.

Supplementary Note 6. Spinless $\mathbf{k}\cdot\mathbf{p}$ model with quadratic corrections

As argued in the main text, one can gain more insight into the main features of the experiment using an effective $\mathbf{k}\cdot\mathbf{p}$ model neglecting the effect of SOC, where the band structure of CoSi has a threefold crossing at Γ and a double Weyl crossing at R. The double Weyl crossing at R has linear

dispersion for all energies of interest, so the relevant model is that of Ref. [2], which is explicitly

$$H_R = \begin{pmatrix} v_R \sigma \cdot \mathbf{k} & 0 \\ 0 & v_F \sigma \cdot \mathbf{k} \end{pmatrix} \tag{S32}$$

The spinless threefold model at Γ has also been presented in Ref. [2] but only to linear order in momentum. For this work, we need to extend it up to quadratic order, which we do next.

CoSi has space group 198, and for effective models near Γ we only need the irreducible representations (irreps) of the point group, which is T. However, it will be useful to first derive the model for the point group of higher symmetry O, and then find the terms that break the symmetry down to T. This will clarify the relative importance of the different quadratic corrections.

The point group O can be generated by C_3 rotations around (111), C_2 rotations around (100) and C_2' rotations around (110), and it has 5 irreps transforming as $A_1 \sim x^2 + y^2 + z^2$, $A_2 \sim xyz$, $E \sim (x^2 - y^2, (2z^2 - x^2 - y^2)/\sqrt{3})$, $T_1 \sim (x, y, z)$ and $T_2 \sim (yz, zx, xy)$. Point group T is obtained by breaking C_2', so it is generated by the C_3 and C_2 operations only, and has three irreps, A, E, T. The subscript in $A_{1,2}$ and $T_{1,2}$ distinguishes the transformation under C_2' in O, and it is absent in T where these pairs of irreps become the same.

The three basis states for the threefold crossing at Γ in O form a T_1 irrep. Operators acting in the subspace of this three basis states can be chosen as the Gell-Mann matrices λ_α with $\alpha = 1, \cdots, 8$ (and $i = 0$ the identity), which are given by

$$\lambda_1 = \begin{pmatrix} 0 & 1 & 0 \\ 1 & 0 & 0 \\ 0 & 0 & 0 \end{pmatrix} \quad \lambda_2 = \begin{pmatrix} 0 & -i & 0 \\ i & 0 & 0 \\ 0 & 0 & 0 \end{pmatrix} \quad \lambda_3 = \begin{pmatrix} 1 & 0 & 0 \\ 0 & -1 & 0 \\ 0 & 0 & 0 \end{pmatrix} \quad \lambda_4 = \begin{pmatrix} 0 & 0 & 1 \\ 0 & 0 & 0 \\ 1 & 0 & 0 \end{pmatrix} \tag{S33}$$

$$\lambda_5 = \begin{pmatrix} 0 & 0 & -i \\ 0 & 0 & 0 \\ i & 0 & 0 \end{pmatrix} \quad \lambda_6 = \begin{pmatrix} 0 & 0 & 0 \\ 0 & 0 & 1 \\ 0 & 1 & 0 \end{pmatrix} \quad \lambda_7 = \begin{pmatrix} 0 & 0 & 0 \\ 0 & 0 & -i \\ 0 & i & 0 \end{pmatrix} \quad \lambda_8 = \frac{1}{\sqrt{3}} \begin{pmatrix} 1 & 0 & 0 \\ 0 & 1 & 0 \\ 0 & 0 & -2 \end{pmatrix} \tag{S34}$$

They transform as bilinears of the basis states so they must form irreps $T_1 \otimes T_1 = A_1 + E + T_1 + T_2$. Taking the standard representation of the rotation operators as

$$C_{3,(111)} = \begin{pmatrix} 0 & 0 & 1 \\ 1 & 0 & 0 \\ 0 & 1 & 0 \end{pmatrix}, \quad C_{2x} = \begin{pmatrix} -1 & 0 & 0 \\ 0 & -1 & 0 \\ 0 & 0 & 1 \end{pmatrix}, \quad C'_{2(110)} = \begin{pmatrix} -1 & 0 & 0 \\ 0 & 0 & 1 \\ 0 & 1 & 0 \end{pmatrix}, \tag{S35}$$

it can be checked that the following combinations of Gell-Matrices transform as the irreps of point group O:

$$T_1 = (-\lambda_2, \lambda_5, -\lambda_7), \tag{S36}$$

$$T_2 = (\lambda_1, \lambda_4, \lambda_6), \tag{S37}$$

$$E = (-\tfrac{1}{2}\lambda_3 + \tfrac{\sqrt{3}}{2}\lambda_8, -\tfrac{\sqrt{3}}{2}\lambda_3 - \tfrac{1}{2}\lambda_8), \tag{S38}$$

while λ_0 trivially transforms as A_1. Time reversal symmetry is implemented as complex conjugation, so $\mathcal{T}^{-1}T_1\mathcal{T} = -T_1$ is odd while $\mathcal{T}^{-1}T_2\mathcal{T} = T_2$ and $\mathcal{T}^{-1}E\mathcal{T} = E$ are even. The matrices in T_1 are also the spin-1 matrices $\mathbf{S}$ used in the main text. The effective Hamiltonian can now be built making scalar combinations of the Gell-Mann matrices with momentum irreps, which up to second order are

$$K_{A1} = k_x^2 + k_y^2 + k_z^2, \tag{S39}$$

$$K_{T1} = (k_x, k_y, k_z), \tag{S40}$$

$$K_{T2} = (k_y k_z, k_x k_z, k_x k_y), \tag{S41}$$

$$K_E = (k_x^2 - k_y^2, (2k_z^2 - k_x^2 - k_y^2)/\sqrt{3}), \tag{S42}$$

which allows four terms in the Hamiltonian preserving time-reversal symmetry

$$H_O = \begin{pmatrix} ak^2 + \dfrac{2c}{3}(k^2 - 3k_z^2) & ivk_x + bk_y k_z & -ivk_y + bk_x k_z \\ -ivk_x + bk_y k_z & ak^2 + \dfrac{2c}{3}(k^2 - 3k_y^2) & ivk_z + bk_x k_y \\ ivk_y + bk_x k_z & -ivk_z + bk_x k_y & ak^2 + \dfrac{2c}{3}(k^2 - 3k_x^2) \end{pmatrix}, \tag{S43}$$

where $k = \sqrt{k_x^2 + k_y^2 + k_z^2}$. If we now consider the physical point group T, a single extra term is allowed because there is a new momentum irrep $K_E' = (-(2k_z^2 - k_x^2 - k_y^2)/\sqrt{3}, k_x^2 - k_y^2)$, which leads to

$$H_T = H_O + \frac{2d}{\sqrt{3}} \begin{pmatrix} k_y^2 - k_x^2 & 0 & 0 \\ 0 & k_x^2 - k_z^2 & 0 \\ 0 & 0 & k_z^2 - k_y^2 \end{pmatrix}. \tag{S44}$$

To obtain the values of this coefficients, we expand the energies of the three bands to lowest order in momentum. The exact energy of any three band Hamiltonian written in terms of the Gell-Mann matrices $H = \lambda_0 h_0 + h_\alpha \lambda_\alpha$ is given by

$$E_n = h_0 + 2\sqrt{\frac{h_2}{3}} \cos\left[\frac{1}{3}\arccos\left(\frac{h_3}{h_2}\sqrt{\frac{3}{h_2}}\right) + \frac{2\pi n}{3}\right] , \tag{S45}$$

where $\alpha = 1, \ldots, 8$, $h_2 = h_\alpha h_\alpha$, $h_3 = d_{\alpha\beta\gamma} h_\alpha h_\beta h_\gamma$, and $d_{\alpha\beta\gamma}$ are the SU(3) symmetric structure constants. Expanding the energies up to second order in k we find

$$E_1 = -vk + ak^2 - \left(b + \frac{2c}{3}\right) f_1(\mathbf{k})k^2 + \frac{2c}{3} f_2(\mathbf{k})k^2, \tag{S46}$$

$$E_2 = ak^2 + 2\left(b + \frac{2c}{3}\right) f_1(\mathbf{k})k^2 - \frac{4c}{3} f_2(\mathbf{k})k^2, \tag{S47}$$

$$E_3 = vk + ak^2 - \left(b + \frac{2c}{3}\right) f_1(\mathbf{k})k^2 + \frac{2c}{3} f_2(\mathbf{k})k^2, \tag{S48}$$

where $k = \sqrt{k_x^2 + k_y^2 + k_z^2}$, $f_1(\mathbf{k}) = \left(k_x^2 k_y^2 + k_y^2 k_z^2 + k_z^2 k_x^2\right)/k^4$ and $f_2(\mathbf{k}) = \left(k_x^4 + k_y^4 + k_z^4\right)/k^4$. We observe that there are no terms proportional to d in this expansion to second order, which means d cannot be obtained by fitting the bands alone. However, this does not mean that we can set d to zero, as the matrix elements of the Hamiltonian still depend on d. In this model, to fit d from ab-initio input would require an ab-initio calculation of the low energy Berry curvature or some other matrix element that is sensitive to d, as it was done in [5]. For our purposes, we leave d as a free parameter, and check that it has a negligible influence on the CPGE. In the main text, for simplicity we present the model with $d = 0$. Inclusion of this term would have only been important to describe effects that are forbidden under O but finite under T, like the linear photo-galvanic effect (LPGE) or second harmonic generation (SHG).

From ab-initio computations, we obtain the value of v, a, b and c by fitting the energy bands near the Γ point. The results to order k^2 are shown in Figs. 9.

We can now proceed to compute the CPGE using Eq. 2 of the main text. For the R point, since we assume a linear double Weyl model with no tilt due to time-reversal, the CPGE has been computed before [2], and it is given by a sharp step function $\theta(\omega - \omega_0)$. The position of the step ω_0 can be estimated, to a very good approximation, by the energy difference in the R-X direction where there is no splitting to any order. When μ crosses the node, we obtain $\omega_0 = 286$ meV, and for $\mu = -10$ meV we obtain $\omega_0 = 267$ meV.

For the Γ point calculation, quadratic corrections have two effects: first, they modify the energies inside the δ function and Fermi functions, modifying the allowed transitions and thus the JDOS. Second, they modify the matrix elements in the integrand, spoiling the perfect quantization of the linear model. In Supplementary Figure 10b we calculate the CPGE for the Γ point considering only the first effect. Because the matrix elements are that of the linear model, the transition $1 \to 3$ is forbidden by angular momentum conservation. For the other two transitions $1 \to 2$ and $2 \to 3$, the effect of quadratic corrections is to modify the energies where they become active or inactive. These critical frequencies are determined by the dispersion in specific directions where the

energy differences between bands are minimal or maximal, for a given modulus of $\mathbf{k}$. For our ab-initio parameters and for sufficiently small negative chemical potential, the directions where energy differences are minimal or maximal are always $\Gamma - X$ and $\Gamma - R$, respectively (see Supplementary Figure 10a). Since bands 1 and 2 are partially filled, there is a frequency window (ω_1^X, ω_1^R) from where the transition $1 \to 2$ activates to where it becomes maximal, and another frequency window (ω_2^X, ω_2^R) where its contribution decreases to zero. On the contrary, band 3 is always empty, so the transition $2 \to 3$ activates in the frequency window (ω_3^X, ω_3^R) and does not disappear at larger frequencies. These critical frequencies can be found analytically to first order in the chemical potential and read

$$\omega_1^X = -\mu\left(1 + \frac{4c-3a}{3v^2}\mu\right) \,, \tag{S49}$$

$$\omega_2^X = \frac{6c}{4c-3a}\mu + v\sqrt{\frac{-3\mu}{4c-3a}} \,, \tag{S50}$$

$$\omega_3^X = \frac{-6c}{4c-3a}\mu + v\sqrt{\frac{-3\mu}{4c-3a}} \,, \tag{S51}$$

$$\omega_1^R = -\mu\left(1 - \frac{2b+3a}{3v^2}\mu\right) \,, \tag{S52}$$

$$\omega_2^R = \frac{3b}{2b+3a}\mu + v\sqrt{\frac{3\mu}{2b+3a}} \,, \tag{S53}$$

$$\omega_3^R = \frac{-3b}{2b+3a}\mu + v\sqrt{\frac{3\mu}{2b+3a}} \,. \tag{S54}$$

In our CoSi effective model with the fitted ab-initio parameters, we find that $\mu = -40$ meV is well within the applicability of these equations. The transition $1 \to 2$ starts to die out before the $2 \to 3$ picks up, which leaves a dip in the CPGE. Note that we have not included ω_2^R and ω_3^R in the main text because they take too large values due to the flatness of the intermediate band. To compute the total CPGE we also need the contribution from the R point, which produces a sharp jump. When this sharp jump accidentally occurs in the middle of the dip contributed by Γ, we generically get a dip-peak structure as observed ab-initio (see Supplementary Figure 10c). For different model parameters, the critical frequencies might be determined by the dispersion in other directions different than $\Gamma - X$ and $\Gamma - R$, and can only be calculated numerically.

Including now the corrections to the integrand (see Supplementary Figure 10d), we observe that they only lead to a smooth change that grows with frequency, without changing the curves qualitatively. Transitions from band 1 to 3 are now allowed, but we found them negligible for our parameter set and were not included in any plot. The origin of the dip can therefore be attributed to the change in JDOS induced by quadratic corrections. The dip remains approximately quantized as it originates from transitions $1 \to 2$ with a closed manifold. In contrast, the peak is generically

non-universal since it is composed from contributions of both the Γ and R. If the frequency window where the Γ contribution vanishes overlaps with the window where the R point transitions contribute, the peak becomes universal.

Finally, we have also studied the effect of the parameter d, which was set to $d = 0$ in Supplementary Figure 10d. We have recomputed all the curves in this figure with $d = 1$ and checked that for $\omega < 0.4$ eV, the curves deviate from those at $d = 0$ by 3% at most, and this difference reduces monotonically for lower energies. As anticipated, the effect of this parameter is negligible in CPGE.

[1] Chang, G. *et al.* Unconventional chiral fermions and large topological fermi arcs in RhSi. *Phys. Rev. Lett.* **119**, 206401 (2017).

[2] Flicker, F. *et al.* Chiral optical response of multifold fermions. *Phys. Rev. B* **98**, 155145 (2018).

[3] Sánchez-Martínez, M.-Á., de Juan, F. & Grushin, A. G. Linear optical conductivity of chiral multifold fermions. *Phys. Rev. B* **99**, 155145 (2019).

[4] Xu, B. *et al.* Optical signatures of multifold fermions in the chiral topological semimetal CoSi. *Proc. Natl. Acad. Sci. U.S.A* **117**, 27104–27110 (2017).

[5] Cook, A. M., Fregoso, B. M., De Juan, F., Coh, S. & Moore, J. E. Design principles for shift current photovoltaics. *Nat. Commun.* **8**, 14176 (2017).
